# The function of “looking-at-nothing” for sequential sensorimotor tasks: Eye movements to remembered action-target locations 

**DOI:** 10.16910/jemr.12.2.2

**Published:** 2019-06-27

**Authors:** Rebecca M. Foerster

**Affiliations:** Center for Interdisciplinary Research (ZiF) & Department of Psychology & Cluster of Excellence ‘Cognitive Interaction Technology’ (CITEC), Germany

**Keywords:** eye movements, looking-at-nothing, saccades, gaze, sensorimotor control, memory, sequence learning, attention

## Abstract

When performing manual actions, eye movements precede hand movements to target locations: Before we grasp an object, we look at it. Eye-hand guidance is even preserved when visual targets are unavailable, e.g., grasping behind an occlusion. This “looking-atnothing” behavior might be functional, e.g., as “deictic pointer” for manual control or as memory-retrieval cue, or a by-product of automatization. Here, it is studied if looking at empty locations before acting on them is beneficial for sensorimotor performance. In five experiments, participants completed a click sequence on eight visual targets for 0-100 trials while they had either to fixate on the screen center or could move their eyes freely. During 50-100 consecutive trials, participants clicked the same sequence on a blank screen with free or fixed gaze. During both phases, participants looked at target locations when gaze shifts were allowed. With visual targets, target fixations led to faster, more precise clicking, fewer errors, and sparser cursor-paths than central fixation. Without visual information, a tiny free-gaze benefit could sometimes be observed and was rather a memory than a motor-calculation benefit. Interestingly, central fixation during learning forced early explicit encoding causing a strong benefit for acting on remembered targets later, independent of whether eyes moved then.

## Introduction

When performing manual actions, such as driving, writing, or grasping an object, we use our eyes to guide our hands to target locations in the environment (1, 2, 3, 4, 5, 6, 7, 8, 9). Eye-hand guidance is important because foveal and thus high resolved visual information about the action target can be extracted during the target fixation and is used to specify hand-movement parameters (6, 10, 11, 12, 13, 14, 15, 16, 17). As a result, hand-movement control is faster and more accurate when target fixations are allowed than when they are omitted (18, 19, 20). However, even in the absence of visual information (e.g., grasping something behind an occlusion), the eyes are sometimes directed to target locations, especially if the sensorimotor task is highly practiced (21, 22) – meaning that the actor is literally looking at nothing. Looking-at-nothing has originally been found during visual imagery and memory-recall tasks (23, 24, 25, 26, 27, 28, 29, 30, 31, 32, 33) and it has been argued that it is functional. Saccading to locations in space that are related to the material that is asked to be retrieved can, for instance, facilitate memory recall (26, 29, 34, 35). Looking at remembered target locations might also constitute a rehearsal process to consolidate memory for later retrieval (36, 37). In the case of motor actions such as reaching, grasping, or pointing, fixations to target locations might additionally serve as “deictic pointers” that facilitate motor calculations (2, 38, 39, 40, 41). Motor calculation might be best if based on the well-learned eye to hand motor transformations based on the efference copy of the eyes (10, 39, 42, 43, 44).

By which mechanism could fixating a target location facilitate hand-movement programming in the absence of visual targets? A single step of a manual action typically consists of a first covert shift of attention, a subsequent eye movement, and a final hand movement to and a manipulation at the action-target location. With visual information, target features such as shape or size can be extracted by a target fixation and used for motor calculation. Without visual information, the fixation point can still be used to locate the remembered target for hand movement calculation in external three-dimensional space, e.g., by calculating the remembered distance of a target to the hand on the basis of the currently fixated point in the world. Neurons that are tuned to zero-disparity at the fovea can be used in the process to connect the internal space of the eyeball position in the head to the external three-dimensional space of the fixation position in the world (38). The idea is that this leads in turn to a fast access of the remembered target location in external space (e.g., 5 cm above fixation in the world) and should facilitate hand-movement programming (45). In addition, while gaze is overtly pointing to the current hand-target location, covert attention can already be newly distributed to subsequent task-related locations. This is possible because attention can be shifted without moving the eyes, contrastingly to the obligatory covert shift of attention preceding each gaze shift (46). In this way, a hand movement can be programmed to the currently fixated target position in parallel with the attentional target-selection process for the subsequent action step. This should fasten the execution of the multiple steps needed for a sensorimotor sequence.

Although it is plausible to assume that looking-at-nothing is functional for sensorimotor control on the basis of the aforementioned reasons, no study has justified any of these assumptions so far. The fact that looking-at-nothing has been found in sensorimotor tasks ( 21, 22, 47) does not prove that this looking-at-nothing behavior is beneficial for sensorimotor performance. An alternative possibility is that looking-at-nothing constitutes a functionless by-product of learning and automatization. As looking at empty target locations does not hamper task execution, it might be applied even if it is not beneficial for task performance just because target locations are usually fixated before acting on them.

### The Present Study

In sum, there are several possible functions of looking-at-nothing in general as well as specifically for sensorimotor tasks which need to be tested. The present study aimed at clarifying which function looking at invisible action-target locations might fulfill. In five experiments, participants performed a computerized adaptation of the number connection test or trail making test, version A (48, 49, 50, 51). In this adaptation of the test, which is a sequential sensorimotor task, participants had to click as fast as possible with a mouse cursor one specific target-location sequence on the computer screen for several trials while they had either to keep central fixation or were allowed to move their eyes freely. Task performance was compared between central fixation and free gaze in terms of task-completion time, click precision, number of erroneous clicks, and the length of the path the cursor moved.

## Experiment 1

### Methods

#### Participants

Participation in all reported experiments followed provision of written informed consent. All experiments were approved by the Committee for Ethics at Bielefeld University (EUB) and performed in accordance with the approved guidelines. All participants reported normal visual acuity or were tested with correcting lenses and were recruited at Bielefeld University, Germany. All were naïve with respect to the purpose of the respective study and were paid for their participation. Forty right-handed students (14 male and 27 female) with a mean age of 24 years completed Experiment 1. Two additional participants did not complete the experiment and did therefore not enter the analyses.

#### Materials

Experimentation took place in a dimly lit room. The experiment was controlled by the Experiment Builder software (SR Research, Ontario, Canada) on a Dell Optiplex 755 computer. The stimuli were displayed on a 19-inch color CRT monitor (ViewSonic Graphics Series G90fB using an ATI Radeon HD 2400 Pro graphics card) with a refresh rate of 100 Hz and a resolution of 1,024 x 768 pixels extending to 36 x 27 cm. The computer mouse and keyboard as well as an extra-large mouse pad (88 x 32 cm) were used. Each participant’s right gaze position was recorded with 1,000 Hz by an EyeLink 1000 tower system (SR Research, Ontario, Canada). Participants’ viewing distance was fixed at 71 cm by the system’s chin and forehead rest throughout the experiment. Color and luminance were measured in CIE Lxy coordinates using an X-Rite i1 Pro spectrophotometer (Munich, Germany).

**Figure 1.**
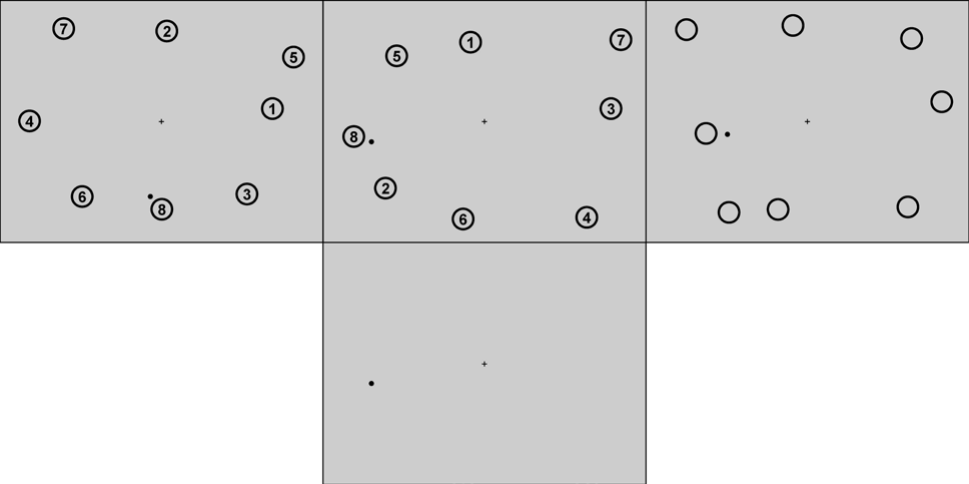
Displays during sequential-clicking in the visual phase (top) of Experiments 1 (left), 2 and 3 (middle), and 4 (right) and in the blank-screen phase of all experiments (bottom).

The computer screen showed a gray background (L = 78.9 cd/m^2^, x = .29, y = .30). A black plus (L = 0.3 cd/m^2^, x = .32, y = .33) of 0.43 degrees of visual angle (°v.a.) in width and height was located in the center of the screen and served as central fixation cross in case of restricted eye movements. A black dot (L = 0.3 cd/m^2^, x = .32, y = .33) with a diameter of approximately 0.43°v.a. constituted the mouse cursor. Eight circular target areas with a diameter of 3.06°v.a. were defined. Their spatial configuration was randomly generated with the prerequisite that each outer field of an imagined 3x3 grid contained one target area, and target areas had a minimal distance of 2.04°v.a. to each other (border-to-border) as well as to the screen border. In the visual-target trials, each target region contained one of eight black numbers in its center (1-8 in Arial, font style bold, font size of 35, which equaled to approximately 0.96°v.a. height and 0.62°v.a. width). Each number was surrounded by an unfilled black circle (2.04°v.a. diameter, line width 6). The target configuration was the same throughout the entire experiment. The stimuli layout can be seen in Figure 1 (upper left).

#### Procedure

The experiment was divided into a first visual-learning phase with numbered circles on the screen and a consecutive blank-screen recall phase. Each phase started with a written instruction on the screen followed by a 9-point calibration and validation procedure.

In the visual-acquisition phase, participants had to click as fast as possible in ascending order on the eight numbered circles presented on the screen (Figure 1, top left). Participants were informed that the configuration of target stimuli stayed the same throughout the whole experiment, so that they could learn the location sequence. In the blank-screen recall phase, only the dot cursor and the central fixation plus were displayed on the grey background (Figure 1, bottom) and participants were instructed to click as fast as possible in the same sequence on the locations which had been occupied by a numbered circle during the visual-acquisition phase. Both phases were preceded by an example trial that was not included in the analysis.

A click was counted as correct within the circular target area (diameter of 3.06°v.a.). A correct click was followed by a high-pitched tone. After all eight target regions were clicked on in the correct order trial-completion time was displayed on the screen. Each trial was preceded by a central fixation on a black ring (0.45°v.a. outer size and 0.11°v.a. inner size) for calibration check. Calibration and validation were repeated if necessary.

The visual-learning phase consisted of 100 trials and the blank-screen recall phase consisted of 50 trials, both performed in blocks of 10 trials. A block information display separated each block. Participants could start each block and trial by pressing the space bar. Participants were allowed to take self-paced breaks in-between blocks and trials.

Half of the participants were instructed to keep fixation on the central plus throughout each visual-learning trial (fix-learning group), while the other half did not receive any instruction concerning their eye movements for the visual-learning phase (free-learning group). During the blank-screen recall phase half of the participants of each learning group had to keep central fixation throughout each trial (fix-recall group), while the other half could use their eyes freely (free-recall group), resulting in two recall groups with counterbalanced learning conditions (FreeFree, FreeFix, FixFree, FixFix). The fixation manipulation during the visual-learning phase was included for two reasons. On the one hand, it enables to verify whether foveal vision benefits acting on visual targets also in the task applied here ‑ a prerequisite for investigating whether the same task also benefits from looking at empty target locations. On the other hand, performance during recall might depend on how the task was learned. It is known that information can be retrieved best when the circumstances are kept constant (cf. encoding specificity: 52, 53). A completely balanced design allows taking this possible correspondence benefit into account.

In the central fixation condition, participants could only start the task when their gaze point was detected within 3.06°v.a. around the central plus. After having started the task, the detection of any fixation outside this central area caused that the trial was abandoned and started from the beginning. Each learning group’s fastest best time during the visual phase as well as each learning x recall group’s fastest best time during recall was awarded with 5 Euros.

#### Analysis

The following performance measures were analyzed as dependent variables: Trial completion time, number of errors, click precision, and cursor-path length. Cursor-path lengths were calculated from 100 Hz display messages as cumulative inter-sample distances per trial using Matlab R2013b (MathWorks, Natick, Massachusetts, USA). Besides the total number of errors, two error types were analyzed. Incorrect clicks that were less than three circle radii distant from the center of the current sequence target were defined as precision errors. Incorrect clicks that were less than three circle radii distant from any other item were defined as sequence errors.

Mixed design analyses of variance (ANOVAs) with groups (free vs. fix learning and free vs. fix recall) as between-subject factors and block (1-10 and 11-15) as within-subject factor were calculated per dependent variable and experimental phase (visual vs. blank) to reveal whether the possibility to move the eyes freely benefitted performance. The resulting values are reported in tables. In case of significant interactions, further analyses (ANOVAs and post-hoc *t*-tests) were calculated to compare experimental groups per block. The overall result pattern can best be grasped from the result figures.

The number of guiding fixations was calculated for the free-gaze trials in order to reveal whether sequential target scanning was applied when participants’ eye movements were unrestricted. A guiding fixation is defined as a fixation on the current target shortly before the action on the target is completed ‑ here a click (cf. 3, 50, 54 and directing fixations in 55). The number of fixations outside the central fixation region was analyzed in order to see how successfully participants could obey the fixation instruction.

SR Research’s default velocity algorithm was used to detect fixations (not a blink, <30°v.a./s velocity and <8,000°v.a./s^2^ acceleration). The recording samples of the last nine trials of subject 16 (free learning and fix recall) and the last 27 trials of subject 37 (central fixation throughout) were not written into the raw data file due to space problems. Therefore, these trials are missing in all gaze analyses as well as in the error type analyses.

Event data post-processing was performed using Matlab R2013b (MathWorks, Natick, Massachusetts, USA) and Microsoft Excel 2010 (Seattle, Washington, USA). All statistical analyses were performed using R3.4.0 (56) and the packages ez (57), plyr (58), and psych (59). Plotting routines of ggplot2 (60) and gridExtra (61) were used.

Violations of sphericity were corrected using the Greenhouse-Geisser *ε.* The uncorrected degrees of freedom are reported together with the Greenhouse-Geisser *ε*. In case of Welch correction for *t*-tests, the corrected degrees of freedom are reported. In case of violation of a normal distribution according to the Kolmogorov-Smirnoff test, results were validated with non-parametric tests where appropriate. Only deviating non-parametric results are reported. A chance level of .05 was applied unless reported otherwise.

### Results

#### Learning Phase

On average 0.5 trials were abandoned before a trial was completed in the central-fixation group due to fixation disengagement. This is a ratio of one abandoned to two completed trials. In other words, participants completed on average two of three trials. The free-learning group performed on average 6.8 guiding fixations, indicating that participants who were allowed to move their eyes fixated most of the eight targets before clicking on them.

**Figure 2.**
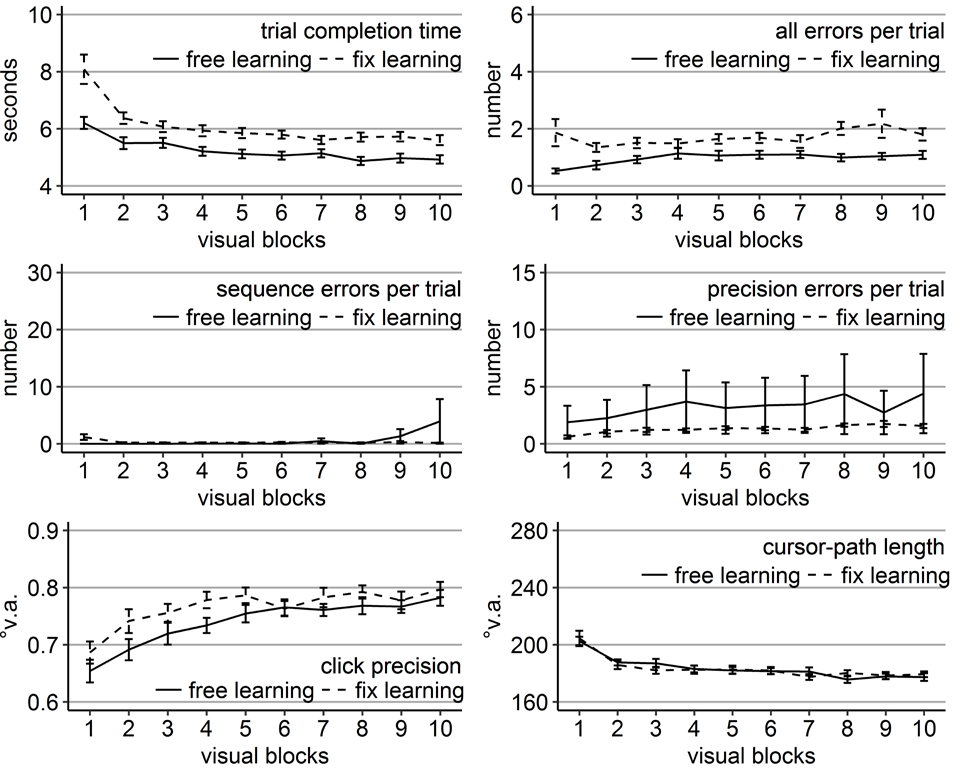
Performance measures during the learning phase of Experiment 1: Trial completion time in seconds, numbers of all errors, sequence errors, and precision errors per trial, click precision in °v.a., and cursor-path length in °v.a. (y-axes) over the course of the ten visual blocks (x-axes) for the two learning groups (free gaze as solid line and fix gaze as dashed line). Error bars represent standard errors of the mean.

The learning group x block ANOVA for trial completion time revealed significant main effects of group and block, as well as a significant interaction (Table 1). Independent sample t-tests revealed that the free-gaze group was significantly faster than the fixation group throughout all visual blocks (ps < 0.05), with more pronounced difference early during learning (Figure 2). The block effect was due to decreasing trial completion time over the course of learning for both groups (linear trend ps < .001).

**Table 1:** ANOVA results of the learning phase of Experiment 1.

***DV***	***effect***	***df***	***F***	***η ^2^***	***p***	***ε***
**completion time**	**L**	**1, 38**	**13.80**	**.18**	**< .001**	
	**B**	**9, 342**	**36.69**	**.27**	**< .001**	**.18**
	**L x B**	**9, 342**	**4.37**	**.04**	**< .05**	**.18**
**number of all errors**	**L**	**1, 38**	**16.12**	**.13**	**< .001**	
	B	9, 342	1.58	.03	.22	
	L x B	9, 342	1.50	.02	.20	
**number of sequence errors**	L	1, 38	0.24	.00	.62	
	B	9, 342	0.95	.02	.49	
	L x B	9, 342	1.14	.02	.33	
**number of precision errors**	L	1, 38	0.64	.02	.43	
	**B** (≠ *Χ ^2 ^*test)	**9, 342**	**2.26**	**.00**	**< .05**	
	L x B	9, 342	0.83	.00	.59	
**click precision**	L	1, 38	2.65	.04	.11	
	**B**	**9, 342**	**21.15**	**.20**	**< .001**	
	L x B	9, 342	1.03	.01	.41	
**cursor-path length**	L	1, 38	<0.01	.01	.98	
	**B**	**9, 342**	**28.74**	**.27**	**< .001**	**.40**
	L x B	9, 342	1.00	.01	.40	

DV = dependent variable, L = learning group, B = block, df = degrees of freedom, F = test value, η^2^ = generalized eta-squared, p = significance value, ε = Greenhouse-Geisser’s epsilon. Significant effects are printed in bold.

The analysis of all errors revealed a significant main effect of group only (Table 1) with a better average performance of the free-gaze group compared to the fixation group (Figure 2). The analysis of errors classified as sequence errors did not reveal any significant effect (Table 1) as participants mostly did not perform any sequence errors during their completed trials. The analysis of errors classified as precision errors did only reveal a significant block effect (Table 1) as well as a group effect according to non-parametric testing (Friedman Χ ^2^(1) = 10, p < .01). Wilcoxon tests indicated significantly less precision errors performed by the fix-gaze group than the free-gaze group during blocks 5 and 8-10 (ps < .05). The block effect was due to an increasing distance from the target center of erroneous clicks in the range of the target for the fix-gaze group (linear trend p < 0.001), but not for the free-gaze group (linear trend p = 0.23).

The analyses of click precision did only reveal a significant block effect, as did the analysis of cursor-path length (Table 1). Cursor-path lengths decreased (linear trend p < .001), while clicking became less centered on the targets (linear trend p < .001) over the course of the learning phase.

#### Recall Phase

**Figure 3.**
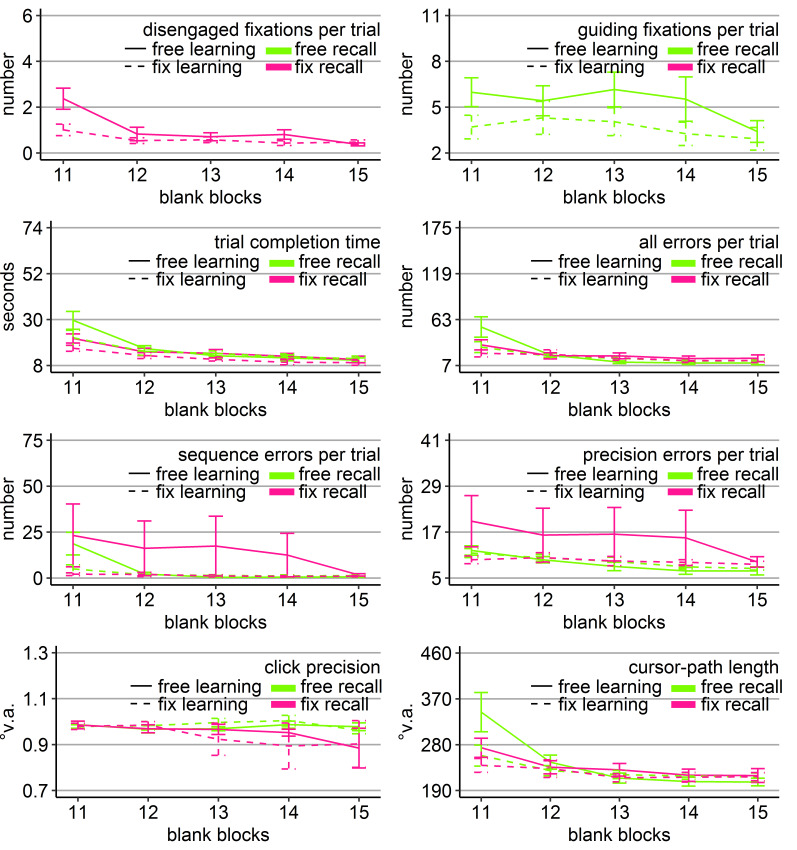
Gaze and performance measures during the recall phase of Experiment 1. The top left plot shows how often participants who were asked to keep central fixation, disengaged their fixation per trial (y-axis) over the course of the five blank blocks (x-axis) depending on whether they learned the clicking sequence with free gaze (solid line) or with fixed gaze (dashed line). The top right plot shows how many guiding fixations participants who were allowed to move their eyes freely performed per trial (y-axis) during the five blank blocks (x-axis) depending on whether they had learned the clicking sequence with free gaze (solid line) or with fixed gaze (dashed line). The six bottom plots show the six performance measures trial completion time in seconds, numbers of all errors, sequence errors, and precision errors per trial, click precision in °v.a., and cursor-path length in °v.a. (y-axes) over the course of the five blank blocks (x-axes) for the four group combinations of recall with free gaze (green lines) or fix gaze (pink lines) after learning with free gaze (solid lines) or fix gaze (dashed lines). Error bars represent standard errors of the mean.

On average 0.7 trials were abandoned before a trial was completed when central fixation was required. Did the frequency of fixation disengagements depend on whether participants had learned with central fixation or free gaze? A mixed-design ANOVA with block (11-15) as within-subject factor and learning group (free vs. fix) as between-subject factor was performed on those 20 participants who had to keep central fixation throughout each recall trial. The analysis revealed a significant block effect, a significant block x group interaction, but no group effect (Table 2). The group that learned with free gaze improved significantly in keeping central fixation over the course of the recall phase (linear trend *p* < .01), while the group that learned already with central fixation showed only a trend towards further improvement (linear trend *p* = .11). The significant block x group interaction was due to the fact that the two learning groups differed significantly in their ability to keep central fixation only during the first recall block (Block 11: *t*(13.99) = 2.59, *Cohen’s d_z_* = 1.16, *p* < .05; Figure 3). Nevertheless, this result indicates how important it is to have both learning groups included to compare performance with gaze restriction against performance with free gaze when acting on an empty screen. A difference between free- and fix- gaze participants that learned with free gaze could likely be due to the fact that the central-fixation group has to learn to keep this central fixation successfully. Comparing the performance of free-recall and fix-recall participants who all learned with central fixation lacks this confound. However, as learning and recall situation is equal only for the fix-learning and fix-recall group, the free-learning and free-recall groups are also required. By a completely matched design, the effects of learning group, recall group, as well as their interaction can be revealed.

**Table 2:** ANOVA results of the recall phase of Experiment 1.

***DV***	***effect***	***df***	***F***	***η ^2^***	***p***	***ε***
**disengage-ments**	L	1, 16	1.59	.05	.23	
	**B**	**4, 64**	**17.06**	**.33**	**< .001**	**.44**
	**L x B**	**4, 64**	**4.99**	**.13**	**< .01**	**.44**
**guiding fixations**	L	1, 18	1.79	.07	.20	
	**B**	**4, 72**	**5.18**	**.05**	**< .01**	**.76**
	L x B	4, 72	1.43	.02	.23	
**completion time**	L	1, 36	2.53	.03	.12	
	R	1, 36	2.81	.04	.10	
	**B**	**4, 144**	**39.79**	**.35**	**< .001**	
	L x R	1, 36	0.23	.00	.63	.43
	**L x B**	**4, 144**	**2.98**	**.04**	**< .05**	
	**R x B**	**4, 144**	**3.25**	**.04**	**< .05**	
	L x R x B	4, 144	1.12	.02	.35	
**number of all errors**	L	1, 36	1.27	.02	.27	
	R	1, 36	0.06	.00	.80	
	**B**	**4, 144**	**24.12**	**.27**	**< .001**	
	L x R	1, 36	0.00	.00	1.00	.39
	**L x B**	**4, 144**	**4.68**	**.07**	**< .01**	**.39**
	**R x B**	**4, 144**	**4.62**	**.07**	**< .01**	**.39**
	L x R x B	4, 144	1.49	.02	.21	
**number of sequence errors**	L	1, 34	3.42	.03	.07	
	R	1, 34	3.33	.03	.08	
	**B**	**4, 136**	**14.27**	**.23**	**< .001**	
	L x R	1, 34	0.38	.00	.54	.27
	**L x B**	**4, 136**	**6.61**	**.12**	**< .001**	**.27**
	**R x B**	**4, 136**	**5.18**	**.10**	**< .001**	**.27**
	L x R x B	4, 136	1.74	.04	.15	
**number of precision errors**	L	1, 34	0.01	.00	.94	
	R	1, 34	0.42	.01	.52	
	**B**	**4, 136**	**17.30**	**.15**	**< .001**	
	L x R	1, 34	0.54	.01	.47	.75
	**L x B**	**4, 136**	**3.51**	**.03**	**< .01**	**.75**
	R x B	4, 136	2.22	.02	.07	.75
	L x R x B	4, 136	0.86	.01	.49	
**click precision**	L	1, 36	0.02	.00	.90	
	R	1, 36	1.63	.02	.21	
	B	4, 144	1.20	.02	.31	
	L x R	1, 36	0.14	.00	.71	
	L x B	4, 144	0.14	.00	.97	
	R x B	4, 144	1.13	.02	.34	
	L x R x B	4, 144	0.45	.01	.77	
**cursor-path length**	L	1, 34	1.80	.03	.19	.33
	R	1, 34	0.71	.01	.41	.33
	**B**	**4, 136**	**26.55**	**.23**	**< .001**	
	L x R	1, 34	0.00	.00	.97	.33
	**L x B**	**4, 136**	**8.82**	**.09**	**< .001**	**.33**
	**R x B**	**4, 136**	**5.28**	**.06**	**< .001**	**.33**
	L x R x B	4, 136	1.17	.01	.33	

DV = dependent variable, L = learning group, R = recall group, B = block, df = degrees of freedom, F = test value, η^2^ = generalized eta-squared, p = significance value, ε = Greenhouse-Geisser’s epsilon. Significant effects are printed in bold.

Averaged over all blocks, free-gaze participants performed 4.5 guiding fixations per recall trial. Did those participants, who were allowed to use their eyes freely, scan the targets similarly irrespective of how they learned the sequence (free vs. fix)? A mixed-design ANOVA for the number of guiding fixations with learning group (free vs. fix) as between-subject factor and blocks (11-15) as within-subject factor was performed on those 20 participants who were allowed to move their eyes freely during recall. The analysis revealed a significant block effect (Table 2, Figure 3). The block effect was due to a decreasing number of guiding fixations over the course of the recall blocks for both groups (linear trend *p*s < .05). Independent sample *t*-tests did not show significant group differences in the number of guiding fixations in any of the five recall blocks (but a trend in Block 11 with *p* = 0.08). Thus, participants scanned the empty target locations sequentially, when they were allowed to move their eyes freely, independently of how they learned the sequence and more so in the beginning of the recall phase.

In order to reveal whether the sequential scanning of empty target locations was beneficial for clicking performance, mixed-design ANOVAs were performed for all performance variables (trial completion time, errors per trial, click precision, and cursor-path length) with block as within-subject factor (11-15), and learning group (free vs. fix) and recall group (free vs. fix) as between-subject factors. The ANOVA for click precision did not reveal any significant effects (Table 2). The ANOVAs for the other performance measures revealed a significant block effect as well as significant block x group interactions, both with learning group and with recall group (except for precision errors that showed only a trend), but neither a learning group x recall group, nor a three-way interaction (Table 2).

Between-subject ANOVAs with learning and recall group per block revealed that the block interactions were due to the fact that groups differed mainly during the first recall block (Block 11). Specifically, only in Block 11, there was a significant main effect of learning group in time (*F*(1, 36) = 4.47, *η ^2^* = .11, *p* < .05), all errors (*F*(1, 36) = 4.80, *η ^2^* = .12, *p* < .05), sequence errors (*F*(1, 36) = 6.49, *η ^2^* = .16, *p* < .05), and path (*F*(1, 36) = 5.88, *η ^2^* = .14, *p* < .05) due to better performance of those participants who learned with central fixation (dotted lines in Figure 3), independent of whether they continued fixating during recall or were allowed to move their eyes. No learning group x recall group interaction reached significance for any of the performance measures in any block. For trial completion time and sequence errors, the main effect of recall group reached significance in Block 11 (time: *F*(1, 36) = 4.90, *η ^2^* = .12, *p* < .05; sequence errors: *F*(1, 36) = 4.86, *η ^2^* = .13, *p* < .05, but Friedman *Χ^2^*(3) = 50, *p* = .27) with faster completion times of those participants who had to keep central fixation (18.60 s vs. 25.46 s), but eventually more sequence errors included (12.63 vs. 11.84).

The block effects of the three-way ANOVA were due to improving performance measures over the course of the recall phase for most of the groups, indicated by linear trends (time: FreeFree *p* < .001, FreeFix *p* < .001, FixFree *p* < .05, FixFix *p* < .001; all errors: FreeFree *p* < .01, FreeFix *p* < .05, FixFree *p* < .05, FixFix *p* = .12; sequence errors: FreeFree *p* < .001, FreeFix *p* < .01, FixFree *p* < .05, FixFix *p* = .62; precision errors: FreeFree *p* < .001, FreeFix *p* < .001, FixFree *p* < .05, FixFix *p* = .77; path: FreeFree *p* < .01, FreeFix *p* < .01, FixFree *p* = .06, FixFix *p* = .36). Only the group that had to keep central fixation throughout the experiment (FixFix) did not improve much over the course of the recall phase, presumably because this group already started the recall phase with near-asymptote performance (Figure 3).

### Discussion

The results of Experiment 1 replicated the finding that visual targets can be acted on faster and more accurately when eye movements are allowed rather than prohibited (18,19,20) and extended this finding to a task without direct target-effector mapping (the hand moves the mouse on the table controlling the cursor on the screen). In addition, it was replicated that when learning a high-speed clicking task, participants become faster, perform less errors, and shorter cursor-paths, but increase their clicking distance to the target center, presumably to achieve the high speed (22). Moreover, it was replicated that participants spontaneously look at target locations shortly before acting on them (guiding fixations) even if no visual information is available (21,22). However, fixating on empty target locations did not result in any performance benefit. Unexpectedly, task performance during the first recall block benefitted from having learned with central fixation, independent of whether eye movements were allowed during recall. This effect seems counterintuitive at first sight. However, because each trial without continuous central fixation was repeated, participants who had to keep central fixation started more trials in total than participants whose gaze was unrestricted. Even if they did not complete those trials, they saw the click configuration longer though only peripherally, possibly producing a practice advantage. In Experiment 2, this group difference was eliminated.

## Experiment 2

In Experiment 2, participants practiced the sequential clicking task with another constant target configuration for 10 blocks of 10 trials each. Again, participants were divided into a central-fixation and a free-gaze group. After the visual-learning phase, participants had to click the learned location sequence on a blank screen for 5 blocks à 10 trials. Half of the participants of each learning group were assigned to the central-fixation condition and the other half to the free-gaze condition during the blank-screen recall phase. In contrast to Experiment 1, fixation disengagement did no longer cause trial abundance and repetition in Experiment 2. Instead, the trial was halted and visual target information was eliminated as soon as an eye sample was detected outside of the fixation region. The trial continued and visual targets reappeared as soon as central fixation was reengaged. In this way, all participants started and completed the same number of trials during both experimental phases and were only exposed to the visual targets while they followed the instruction.

### Methods

A new sample of forty right-handed students (13 male and 27 female) with a mean age of 23 years completed Experiment 2. The data of three additional participants were incomplete and did therefore not enter the analyses.

Apparatus and stimuli were the same as in Experiment 1, only that a new spatial configuration of eight target regions was generated for Experiment 2 (Figure 1, top middle) with the same prerequisites as in Experiment 1. This spatial configuration of target regions was the same throughout the entire experiment.

The procedure was the same as in Experiment 1 except for fixation disengagement handling. When eye movements were restricted and a gaze sample was detected outside of a diameter of 3.06°v.a. around the central plus, the trial was halted. Specifically, a deep tone sounded, the fixation plus turned red, clicks did no longer count, and the cursor was extinguished as were the numbered circles in case of the visual phase. As soon as a gaze sample was detected within the central fixation region again, the trial continued. Specifically, the fixation plus turned black again, clicks counted again, the cursor reappeared as well as the numbered circles in case of the visual phase. In this way, the number of trials that were started and completed was exactly the same for all participants and participants were exposed to the visual material only while they obeyed the instruction.

Analyses were the same as in Experiment 1.

### Results

#### Learning Phase

Central-fixation participants looked on average 1.5 times per trial outside of the central-fixation region. Participants who were allowed to move their eyes freely executed on average 7.0 guiding fixations, indicating that they scanned most of the eight targets before clicking.

Mixed measures ANOVAs with learning group (free vs. fix) as between-subject and block (1-10) as within-subject factor for all four performance measures were conducted to reveal possible benefits of moving the eyes freely.

The analysis of trial completion time mirrored the results of Experiment 1 with significant block and group main effects as well as a significant interaction (Table 3).

Also, the analysis of all types of errors resulted in significant main effects of block and group as well as a significant interaction this time as did the analysis of cursor-path length (Table 3).

Identical to the results of Experiment 1, the ANOVA of click precision yielded only a significant block main effect (Table 3) due to increasingly less central clicking on the targets (linear trend *p* < 001) as well as a trend towards an interaction.

**Table 3:** ANOVA results of the learning phase of Experiment 2.

***DV***	***effect***	***df***	***F***	***η ^2^***	***p***	***ε***
**completion time**	**L**	**1, 38**	**47.72**	**.32**	**< .001**	
	**B**	**9,342**	**56.03**	**.48**	**< .001**	**.15**
	**L x B**	**9, 342**	**37.96**	**.38**	**< .001**	**.15**
**number of all errors**	**L**	**1, 38**	**23.44**	**.21**	**< .001**	
	**B**	**9, 342**	**5.39**	**.07**	**< .001**	**.18**
	**L x B**	**9, 342**	**9.60**	**.12**	**< .001**	**.18**
**number of sequence errors**	**L**	**1, 38**	**29.82**	**.12**	**< .001**	
	**B**	**9, 342**	**16.41**	**.26**	**< .001**	**.14**
	**L x B**	**9, 342**	**16.61**	**.26**	**< .001**	**.14**
**number of precision errors**	**L**	**1, 38**	**8.05**	**.14**	**< .01**	
	**B**	**9, 342**	**21.19**	**.12**	**< .001**	**.56**
	**L x B**	**9, 342**	**4.12**	**.03**	**< .001**	**.56**
**click precision**	L	1, 38	0.07	.00	.80	
	**B**	**9, 342**	**40.93**	**.30**	**< .001**	**.55**
	L x B	9, 342	1.73	.02	.08	
**cursor-path length**	**L**	**1, 38**	**40.01**	**.26**	**< .001**	
	**B**	**9, 342**	**58.56**	**.50**	**< .001**	**.17**
	**L x B**	**9, 342**	**22.29**	**.28**	**< .001**	**.17**

DV = dependent variable, L = learning group, B = block, df = degrees of freedom, F = test value, η^2^ = generalized eta-squared, p = significance value, ε = Greenhouse-Geisser’s epsilon. Significant effects are printed in bold.

**Figure 4.**
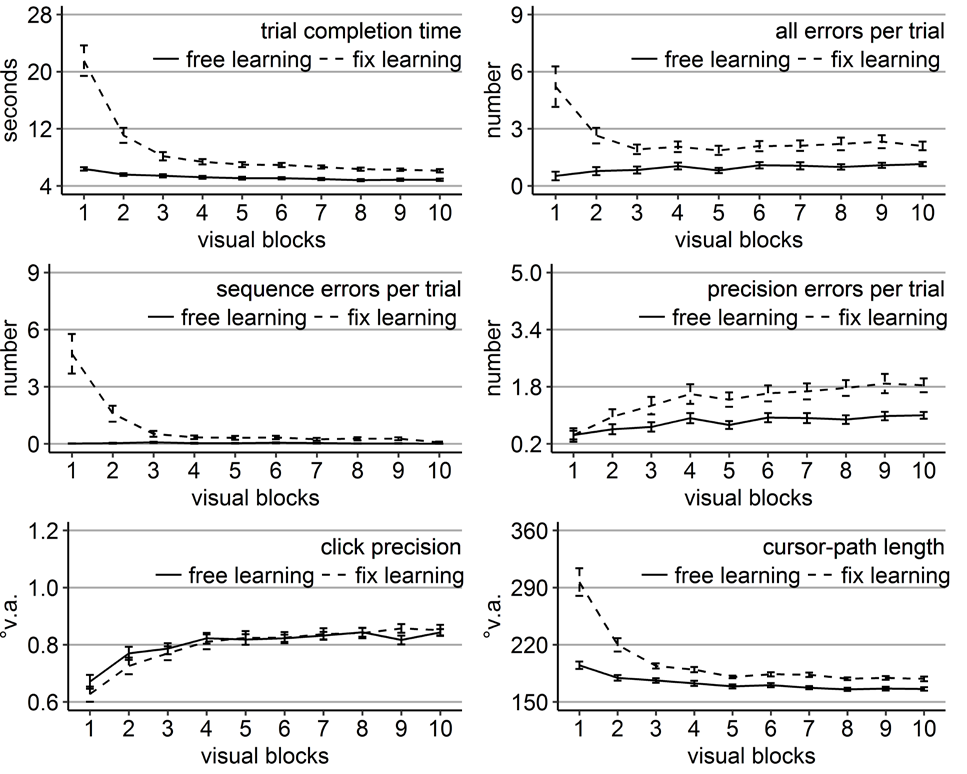
Performance measures during the learning phase of Experiment 2: Trial completion time in seconds, numbers of all errors, sequence errors, and precision errors per trial, click precision in °v.a., and cursor-path length in °v.a. (y-axes) over the course of the ten visual blocks (x-axes) for the two learning groups (free gaze as solid line and fix gaze as dashed line). Error bars represent standard errors of the mean.

Independent sample *t*-tests revealed that the free-gaze participants completed the trials significantly faster, with less incorrect clicks due to both less sequence errors and less precision errors, and with shorter cursor-paths than the fix-gaze participants in all visual blocks (except for precision errors in blocks 1 and 2), with more pronounced differences early during learning (Figure 4).

Most performance measures improved significantly over the course of learning for both groups (linear trend *p*s < .05 for number of errors and *p*s < .001 for all others) except for the number of sequence errors of the free-gaze group (linear trend *p* = .13), which did not perform sequence errors from the beginning. Note, that in contrast to Experiment 1, the fix-gaze participants performed initially a high number of sequence errors in the present study. It is likely that the true number of sequence errors in Experiment 1 was covered due to the procedure of abandoning trials after fixation disengagement that did not enter the analysis.

#### Recall Phase

**Figure 5.**
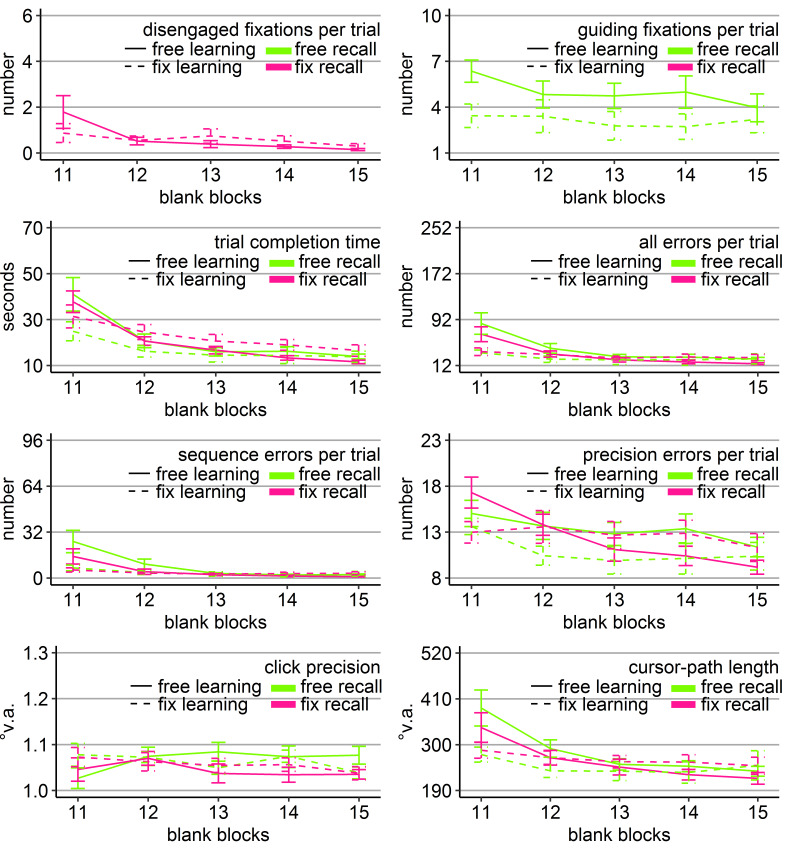
Gaze and performance measures during the recall phase of Experiment 2. The top left plot shows how often participants, who were asked to keep central fixation, disengaged their fixation per trial (y-axis) over the course of the five blank blocks (x-axis) depending on whether they learned the clicking sequence with free gaze (solid line) or with fixed gaze (dashed line). The top right plot shows how many guiding fixations participants, who were allowed to move their eyes freely, performed per trial (y-axis) during the five blank blocks (x-axis) depending on whether they had learned the clicking sequence with free gaze (solid line) or with fixed gaze (dashed line). The six bottom plots show the performance measures trial completion time in seconds, numbers of all errors, sequence errors, and precision errors per trial, click precision in °v.a., and cursor-path length in °v.a. (y-axes) over the course of the five blank blocks (x-axes) for the four group combinations of recall with free gaze (green lines) or fix gaze (pink lines) after learning with free gaze (solid lines) or fix gaze (dashed lines). Error bars represent standard errors of the mean.

During the recall phase, on average 0.6 disengaged fixations were observed per trial when participants were instructed to keep central fixation. A mixed ANOVA with block (11-15) as within-subject factor and learning group (free vs. fix) as between-subject factor revealed only a significant main effect of block, but not of group, nor a significant interaction (Figure 5, Table 4). Thus, the number of disengaged fixations did not differ significantly between the participants who learned with fix vs. free gaze in this experiment with an equal number of started and completed learning trials. The block effect was due to a trend towards improvement in keeping central fixation over the course of the recall phase (linear trend *p* = .05).

Free-gaze participants performed on average 4 guiding fixations per recall trial. A learning group (free vs. fix) by block (11-15) ANOVA revealed a significant block effect and trends towards a group effect and towards a block x group interaction (Table 4). The interaction trend was due to the fact that the learning groups differed significantly in the number of their guiding fixations during the first recall block, but no longer thereafter (Block 11; *t*(17.95) = 2.76, *Cohen’s d_z_* = 1.23, *p* < .05; Figure 5). In addition, only the free-learning group reduced the amount of guiding fixations over the course of the recall phase (linear trend *p* < .05 for free vs. *p* = 0.55 for fix learning) and in this way approached the fix-learning group’s level.

**Table 4:** ANOVA results of the recall phase of Experiment 2.

***DV***	***effect***	***df***	***F***	***η ^2^***	***p***	***ε***
**disengagements**	L	1, 18	0.01	.00	.92	
	**B**	**4, 72**	**5.29**	**.15**	**< .001**	**.32**
	L x B	4, 72	1.92	.06	.12	
**guiding fixations**	L	1, 18	2.67	.11	.12	
	**B**	**4, 72**	**2.83**	**.03**	**< .05**	
	L x B	4, 72	1.79	.02	.14	
**completion time**	L	1, 36	0.22	.00	.65	.36
	R	1, 36	0.63	.01	.43	
	**B**	**4, 144**	**55.26**	**.35**	**< .001**	
	L x R	1, 36	1.97	.03	.17	.36
	**L x B**	**4, 144**	**7.08**	**.07**	**< .001**	**.36**
	R x B	4, 144	0.63	.01	.64	
	L x R x B	4, 144	0.21	.00	.93	
**number of all errors**	L	1, 36	2.26	.04	.14	.32
	R	1, 36	0.26	.00	.61	
	**B**	**4, 144**	**29.70**	**.24**	**< .001**	
	L x R	1, 36	1.45	.02	.24	.32
	**L x B**	**4, 144**	**13.21**	**.12**	**< .001**	**.32**
	R x B	4, 144	0.37	.00	.83	
	L x R x B	4, 144	0.24	.00	.92	
**number of sequence errors**	L	1, 36	3.36	.04	.07	.28
	R	1, 36	1.43	.02	.24	
	**B**	**4, 144**	**19.56**	**.24**	**< .001**	
	L x R	1, 36	1.25	.01	.27	.28
	**L x B**	**4, 144**	**9.19**	**.13**	**< .001**	**.28**
	R x B	4, 144	1.37	.02	.25	
	L x R x B	4, 144	0.66	.01	.62	
**number of precision errors**	L	1, 36	0.87	.02	.36	
	R	1, 36	0.17	.00	.68	
	**B**	**4, 144**	**11.91**	**.11**	**< .001**	**.69**
	L x R	1, 36	1.51	.03	.23	.69
	L x B	4, 144	2.07	.02	.09	
	R x B	4, 144	0.88	.01	.48	
	**L x R x B**	**4, 144**	**3.24**	**.03**	**< .05**	**.69**
**click precision**	L	1, 36	0.10	.00	.75	
	R	1, 36	1.35	.02	.25	
	B	4, 144	1.15	.02	.33	
	L x R	1, 36	0.48	.01	.49	
	L x B	4, 144	2.14	.03	.07	
	R x B	4, 144	0.86	.01	.49	
	L x R x B	4, 144	1.06	.02	.38	
**cursor-path length**	L	1, 36	0.79	.02	.38	.37
	R	1, 36	0.02	.00	.90	
	**B**	**4, 144**	**26.68**	**.18**	**< .001**	
	L x R	1, 36	1.21	.02	.28	.37
	**L x B**	**4, 144**	**9.43**	**.07**	**< .001**	**.37**
	R x B	4, 144	0.66	.01	.62	
	L x R x B	4, 144	0.42	.00	.79	

DV = dependent variable, L = learning group, R = recall group, B = block, df = degrees of freedom, F = test value, η^2^ = generalized eta-squared, p = significance value, ε = Greenhouse-Geisser’s epsilon. Significant effects are printed in bold.

In order to reveal whether the sequential scanning of empty target locations was beneficial for clicking performance, mixed-design ANOVAs were performed for all performance variables with block as within-subject factor (11-15) and learning and recall group (free vs. fix) as between-subject factors. Mirroring the results of Experiment 1, the analysis of click precision did not reveal any significant effects (Table 4), however this time with a trend for the block x learning group interaction. The analyses for the other performance measures revealed again significant block effects and block x learning group interactions or a trend towards it in case of precision errors. However, there was no significant effect of recall group, nor any significant interaction with it (Table 4). The learning group effect showed a trend towards significance only when considering the sequence errors. The three-way interaction reached significance only for the number of precision errors.

Similarly to Experiment 1, between-subject ANOVAs with learning and recall group per block revealed that the block interactions were due to the fact that groups differed significantly only during the first recall block (Block 11). Specifically, there was a main effect of learning group in all five variables (time: *F*(1, 36) = 4.28, *η^2^* = .11, *p* < .05; all errors: *F*(1, 36) = 11.40, *η^2^* = .24, *p* < .01; sequence errors: *F*(1, 36) = 8.16, *η^2^* = .18, *p* < .01; precision errors: *F*(1, 36) = 4.67, *η^2^* = .11, *p* < .05; path: *F*(1, 36) = 7.22, *η^2^* = .17, *p* < .05) due to better performance of the participants that learned with central fixation (dotted lines in Figure 5). Thus, again participants who had learned with central fixation outperformed participants who had learned with free gaze during the first recall block, and that was the case independent of whether or not participants moved their eyes during recall. No learning group x recall group interaction reached significance for any of the performance measures in any block, neither did any recall group main effect.

The block effects of the three-way ANOVAs were mostly due to performance improvements over the course of the recall phase of both learning groups, indicated by linear trends (time: *p*s < .001; all errors: *p*s < .01; sequence errors: *p* < .04; precision errors: *p* < .001 for free learning group and *p* = .10 for the fix learning group; path: *p* < .001 for the free learning group and *p* = 0.08 for the fix learning group).

### Discussion

The results of Experiment 2 replicated the free-gaze benefit for acting on visual targets as well as the finding of looking-at-nothing when performing a well-practiced sensorimotor task in the absence of visual targets (guiding fixations). Again, looking-at-nothing was not accompanied by any performance benefit. Although the learning groups now started and completed the same amount of trials, participants who learned the task with central fixation during the visual phase still outperformed those participants who learned with free gaze when it came to clicking the learned sequence on a blank-screen (Block 11). Specifically, fix-learning participants completed the trials faster with fewer errors of all types and shorter cursor-paths. Importantly, this “fixation-learning” benefit was observed not only for participants who had to continue central fixation, but also for participants who were allowed to move their eyes during recall. The source of this “fixation-learning” benefit will be investigated in Experiment 4. This “fixation-learning” benefit might have covered a potential free-gaze benefit in the blank blocks. Experiment 3 was conducted to investigate whether the recall groups might start to differ at the end of an even longer blank-screen phase, when the “fixation-learning” benefit might no longer exert any coverings.

## Experiment 3

In Experiment 3, the blank-screen phase was extended to 10 blocks of 10 trials each. A new sample of 40 participants was recruited and participants were assigned to a central-fixation and a free-gaze group in the same way as in the previous experiments. Experiment 3 tested whether a benefit of looking-at-nothing can be observed after the “fixation-learning” benefit has completely washed out over the course of a long blank-screen recall phase.

### Methods

A new sample of forty right-handed students (16 male and 24 female) with a mean age of 25 years completed Experiment 3. The data of two additional participants were incomplete and did therefore not enter the analyses.

Apparatus and stimuli were the same as in Experiment 2, including the spatial configuration of the eight target regions (Figure 1, top middle).

The procedure was the same as in Experiment 2, except that the recall phase was extended from 5 to 10 blocks à 10 trials. In this way, it can be measured whether a free-gaze benefit shows up late during the blank-screen recall phase, when the benefit of having learned the configuration with central fixation might have already completely ceased.

Analyses were the same as in the previous experiments.

### Results

#### Learning Phase

Participants who were asked to keep central fixation performed on average 1.6 fixations outside the fixation region. On average 8.2 guiding fixations were executed by participants who were allowed to move their eyes freely, indicating sequential target scanning.

Again, mixed measures ANOVAs (learning group x block) were conducted for all performance measures. Mirroring the results of Experiment 2, the analysis of trial completion time, number of all error types, and cursor-path length resulted in significant block and group main effects as well as significant interactions (Table 5). The analysis of click precision revealed besides a significant block effect also a significant group effect this time, and a trend towards an interaction (Table 5).

**Table 5:** ANOVA results of the learning phase of Experiment 3.

***DV***	***effect***	***Df***	***F***	***η ^2^***	***p***	***ε***
**completion time**	**L**	**1, 38**	**28.18**	**.18**	**< .001**	
	**B**	**9, 342**	**32.28**	**.37**	**< .001**	**.18**
	**L x B**	**9, 342**	**20.50**	**.27**	**< .001**	**.18**
**number of all errors**	**L**	**1, 38**	**35.78**	**.27**	**< .001**	
	**B**	**9, 342**	**5.32**	**.08**	**< .001**	**.21**
	**L x B**	**9, 342**	**10.30**	**.14**	**< .001**	**.21**
**number of sequence errors**	**L**	**1, 38**	**17.84**	**.12**	**< .001**	
	**B**	**9, 342**	**14.29**	**.21**	**< .001**	**.19**
	**L x B**	**9, 342**	**14.37**	**.21**	**< .001**	**.19**
**number of precision errors**	**L**	**1, 38**	**18.28**	**.26**	**< .001**	
	**B**	**9, 342**	**22.14**	**.14**	**< .001**	**.55**
	**L x B**	**9, 342**	**3.30**	**.02**	**< .001**	**.55**
**click precision**	**L**	**1, 38**	**11.11**	**.14**	**< .01**	
	**B**	**9, 342**	**38.96**	**.32**	**< .001**	**.60**
	L x B	9, 342	1.68	.02	.09	
**cursor-path length**	**L**	**1, 38**	**11.30**	**.11**	**< .01**	
	**B**	**9, 342**	**43.32**	**.40**	**< .001**	**.18**
	**L x B**	**9, 342**	**11.73**	**.16**	**< .001**	**.18**

DV = dependent variable, L = learning group, B = block, df = degrees of freedom, F = test value, η^2^ = generalized eta-squared, p = significance value, ε = Greenhouse-Geisser’s epsilon. Significant effects are printed in bold.

**Figure 6.**
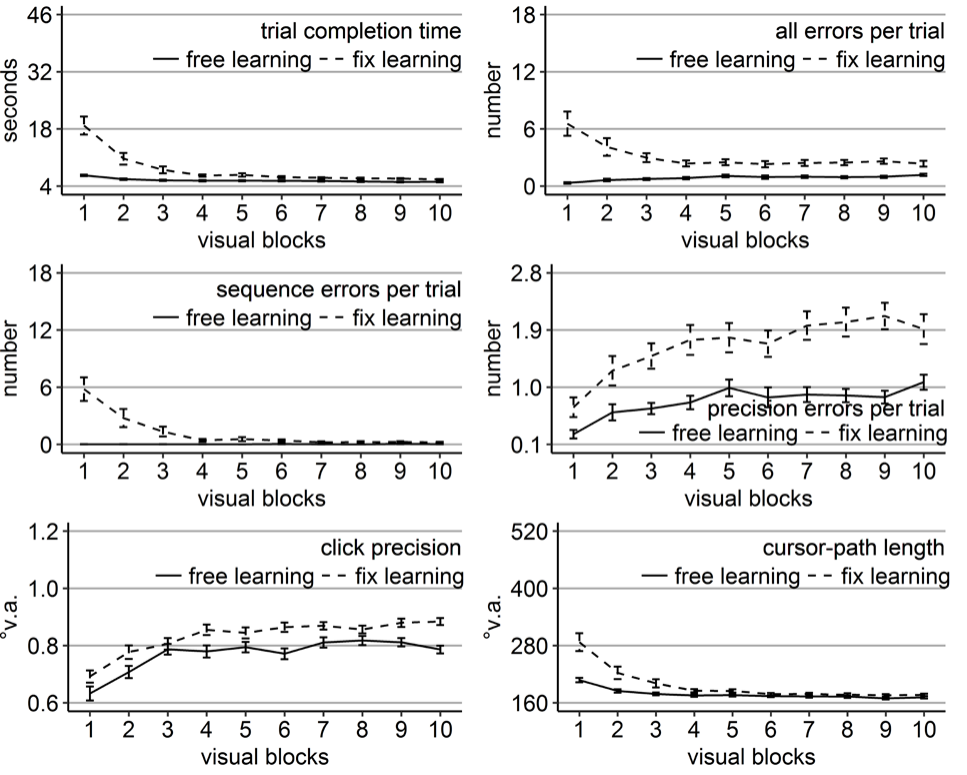
Performance measures during the learning phase of Experiment 3: Trial completion time in seconds, numbers of all errors, sequence errors, and precision errors per trial, click precision in °v.a., and cursor-path length in °v.a. (y-axes) over the course of the ten visual blocks (x-axes) for the two learning groups (free gaze as solid line and fix gaze as dashed line). Error bars represent standard errors of the mean.

Again, free-gaze participants completed a trial significantly faster with fewer errors of all types (*p*s < 0.05, except for sequence errors in block 9 with *p* = 0.06) as confirmed by significant independent sample *t*-tests. Significantly shorter cursor-paths of the fix-gaze participants were observed in the first five blocks (*p*s < 0.05). This time, also click precision of the free-gaze participants was higher in some blocks (*p*s < 0.05 in blocks 2, 4, 6, 7, 9, and 10). Differences were most pronounced early during the learning phase (Figure 6).

Replicating the results of Experiments 1 and 2, time, errors, and cursor-path length improved significantly over the course of learning (linear trend *p*s < .001 for time and path and for errors of the free-gaze group, *p* < .01 for errors of the fix-gaze group, but *p* = .28 for the sequence errors of the free-gaze group), while click precision decreased (linear trend *p*s < .001).

#### Recall Phase

**Figure 7.**
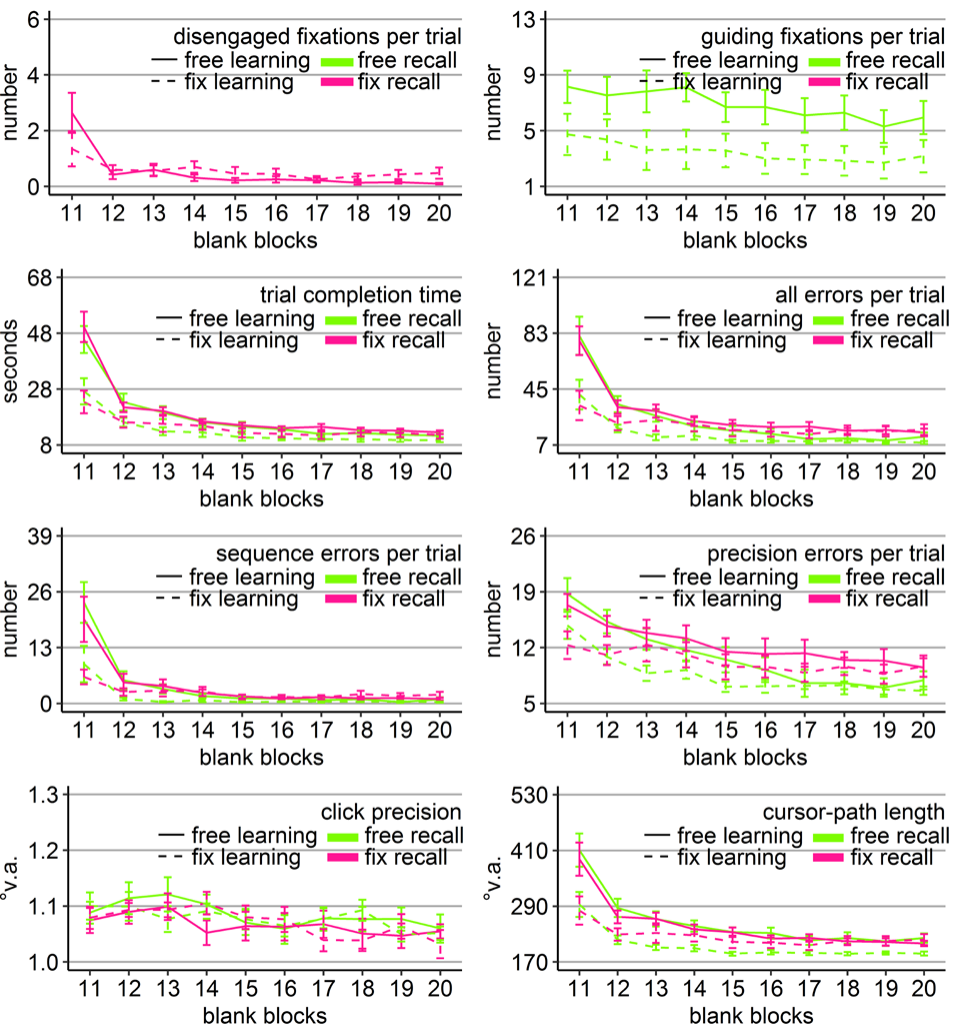
Gaze and performance measures during the recall phase of Experiment 3. The top left plot shows how often participants, who were asked to keep central fixation, disengaged their fixation per trial (y-axis) over the course of the ten blank blocks (x-axis) depending on whether they learned the clicking sequence with free gaze (solid line) or with fixed gaze (dashed line). The top right plot shows how many guiding fixations participants, who were allowed to move their eyes freely, performed per trial (y-axis) during the ten blank blocks (x-axis) depending on whether they had learned the clicking sequence with free gaze (solid line) or with fixed gaze (dashed line). The six bottom plots show the six performance measures trial completion time in seconds, numbers of all errors, sequence errors, and precision errors per trial, click precision in °v.a., and cursor-path length in °v.a. (y-axes) over the course of the ten blank blocks (x-axes) for the four group combinations of recall with free gaze (green lines) or fix gaze (pink lines) after learning with free gaze (solid lines) or fix gaze (dashed lines). Error bars represent standard errors of the mean.

During the recall phase, on average 0.5 fixations were detected outside the fixation region per trial and fix-gaze participant. A mixed ANOVA (learning group x block) revealed a significant block effect and a significant block x group interaction (Figure 7 and Table 6). The effects were due to the fact that disengagements decreased more strongly during recall for the group that did not already learn with central fixation (linear trend of *p* < .01 for the free-learning group, *p* = .06 for the fix-learning group).

Free-gaze participants performed on average 5.2 guiding fixations per blank-screen recall trial. The mixed ANOVA (learning group x block) revealed a significant block effect, a significant group effect, but no significant interaction (Table 6). Linear trend analysis showed eventually tendencies towards a decreasing number of guiding fixations in both groups (linear trend *p* = .11 for the free-learning group and *p* = .17 for the fix-learning group). Learning groups differed significantly during blocks 14 (*t*(16.37) = 2.56, *Cohen’s d_z_* = 1.14, *p* < .05), 16 (*t*(17.77) = 2.22, *Cohen’s d_z_* = .99, *p* < .05), and 18 (*t*(17.61) = 2.12, *Cohen’s d_z_* = .95, *p* < .05) with less guiding fixations of the group that learned with central fixation.

**Table 6:** ANOVA results of the recall phase of Experiment 3.

***DV***	***effect***	***df***	***F***	***η ^2^***	***p***	***ε***
**disengage-ments**	L	1, 18	.08	.00	.79	
	**B**	**9, 162**	**12.13**	**.30**	**< .001**	**.17**
	**L x B**	**9, 162**	**2.65**	**.08**	**< .01**	
**guiding fixations**	**L**	**1, 18**	**4.84**	**.17**	**< .05**	
	**B**	**9, 162**	**3.31**	**.04**	**< .01**	**.23**
	L x B	9, 162	.45	.01	.91	
**completion time**	**L**	**1, 36**	**13.07**	**.15**	**< .001**	**.19**
	R	1, 36	.73	.01	.40	
	**B**	**9, 324**	**100.93**	**.60**	**< .001**	
	L x R	1, 36	.01	.00	.94	.19
	**L x B**	**9, 324**	**17.76**	**.21**	**< .001**	**.19**
	R x B	9, 324	.33	.00	.96	
	L x R x B	9, 324	1.06	.02	.39	
**number of all errors**	**L**	**1, 36**	**7.16**	**.08**	**< .05**	**.18**
	R	1, 36	2.01	.03	.16	
	B	9, 324	58.99	.47	**< .001**	
	L x R	1, 36	.14	.00	.71	.18
	**L x B**	**9, 324**	**11.88**	**.15**	**< .001**	**.18**
	R x B	9, 324	1.18	.02	.31	
	L x R x B	9, 324	.31	.00	.97	
**number of sequence errors**	**L**	**1, 36**	**6.63**	**.05**	**< .05**	**.15**
	R	1, 36	.47	.00	.50	
	**B**	**9, 324**	**36.97**	**.43**	**< .001**	
	L x R	1, 36	.58	.00	.45	.15
	**L x B**	**9, 324**	**9.86**	**.17**	**< .001**	**.15**
	R x B	9, 324	1.09	.02	.37	
	L x R x B	9, 324	.06	.00	1.00	
**number of precision errors**	L	1, 36	3.96	.07	.05	.44
	R	1, 36	2.20	.04	.15	.44
	**B**	**9, 324**	**32.24**	**.25**	**< .001**	
	L x R	1, 36	.01	.00	.91	.44
	**L x B**	**9, 324**	**2.48**	**.02**	**< .01**	**.44**
	**R x B**	**9, 324**	**2.99**	**.03**	**< .01**	**.44**
	L x R x B	9, 324	.75	.01	.66	
**click precision**	**L**	1, 36	.01	.00	.92	
	R	1, 36	.67	.01	.42	
	**B**	**9, 324**	**5.00**	**.06**	**< .001**	
	L x R	1, 36	.11	.00	.74	.68
	L x B	9, 324	.89	.01	.53	
	R x B	9, 324	.78	.01	.63	
	L x R x B	9, 324	1.07	.01	.38	
**cursor-path length**	**L**	**1, 36**	**11.30**	**.15**	**< .01**	**.18**
	R	1, 36	.37	.01	.55	
	**B**	**9, 324**	**68.35**	**.46**	**< .001**	
	L x R	1, 36	1.64	.02	.21	.18
	**L x B**	**9, 324**	**9.14**	**.10**	**< .001**	**.18**
	R x B	9, 324	.92	.01	.51	
	L x R x B	9, 324	.29	.00	.98	

DV = dependent variable, L = learning group, R = recall group, B = block, df = degrees of freedom, F = test value, η^2^ = generalized eta-squared, p = significance value, ε = Greenhouse-Geisser’s epsilon. Significant effects are printed in bold.

Mixed ANOVAs (block x learning group x recall group) were conducted to reveal whether a benefit of sequential scanning of empty target locations could be found with the prolonged recall phase (10 blocks). The analysis for click precision did only reveal a significant block effect (*F*(9, 324) = 5.00, *η^2^* = .06, *p* < .001) due to a slight further decrease in click precision over the course of the recall phase (linear trend *p* < .05; Figure 7). Mirroring the results of Experiment 2, the other performance measures all revealed a significant block effect, a significant learning group effect or at least a trend (precision errors), and a significant learning group x block interaction (Table 6 and Figure 7). Recall group was significant only for the number of sequence errors. None of the analyses delivered a significant three-way interaction.

The learning group x block interactions were again due to the fact that learning groups differed mainly early during the blank-screen recall phase. Specifically, there were significant main effects of learning group in blocks 11-17 for trial completion time and cursor-paths length, in blocks 11-13 for all errors, and in blocks 11 and 12 for sequence and precision errors due to better performance of those participants, who learned with central fixation (Figure 7), independent of whether or not participants moved their eyes during the recall phase.

Although there were no significant effects of recall group and mostly no significant interactions with it, numerically, the free-recall group was slightly better in terms of time, errors, and cursor-paths during later blocks (Figure 7). Indeed, the ANOVAs per block, which were conducted in order to get insights into the learning-group effects (see above), revealed some significant recall-group effects (time in block 17, errors in blocks 17-20, sequence errors in blocks 15, 17, 19, and 20, and precision errors in blocks 18-20) with better performance of the free-recall group late during the recall phase. Additionally, significant recall group x block interactions were found for cursor-paths length in blocks 18 and 20 with significantly better performance of the group that learned with central fixation, but was then allowed to use the eyes freely (Figure 7) compared to all other group combinations that did not differ significantly from each other. These effects need to be interpreted with caution because the three-way ANOVAs did only reveal a significant recall group x block effect for precision errors.

The block effects of the three-way ANOVAs for time, errors, and paths were again due to improvement over the course of learning, indicated by linear trends (time: *p*s < .001; error: *p*s < .001; sequence errors: *p* = .06 for the FixFree and *p*s < .05 for all other combinations; precision errors: *p*s < .05; path: *p*s < .001).

### Discussion

Experiment 3 again replicated the free-gaze benefit for acting on visual targets and the finding of spontaneous fixations of empty target locations. In addition, an exploratory cross-over effect could be observed over the course of the blank-screen phase. During the early blank-screen blocks, the “fixation learning” benefit could be replicated. Specifically, participants who had learned with central fixation outperformed participants who had learned with free gaze when both had to act on a blank screen after learning. This benefit was independent of whether or not participants had to keep central fixation during the recall phase. During the late blank-screen blocks, numerically a small free-gaze benefit arose, i.e., participants who were allowed to complete the task with free gaze outperformed those participants who had to keep central fixation during clicking on the empty target locations. This latter benefit of looking-at-nothing was independent from the learning condition for most of the performance measures. However, the free-gaze benefit during the late blank blocks was much smaller than the enormous “fixation-learning” benefit in the early blank blocks and did not reach significance in the initial three-way interaction, except for the precision errors. It is possible that the effects counteract each other even throughout the long blank-screen phase, so that the real benefit of looking-at-nothing might still have been covered by the “fixation-learning” benefit. Therefore, an experiment is required that eliminates the “fixation-learning” benefit completely. In order to design such an experiment, it is important to understand the reason for the “fixation-learning” benefit.

Why is a learned clicking sequence reproduced more accurately and faster on a blank screen, when the sequence was learned with central fixation rather than with unrestricted eye movements and thus target fixations? When having to keep central fixation, the targets have to be located and identified from peripheral vision. While localization is quite good in the periphery, object identification is restricted in the periphery due to its low resolution (627, 63,64). In the reported experiments, it is hard to identify the 0.96°v.a small numbers in the periphery with a mean distance of 10.5°v.a. from central fixation. Therefore, the central-fixation group has to learn from trial and error with the help of the auditory feedback. This is indicated by the huge amount of sequence errors of those participants who are not allowed to gaze at the numbers. As they cannot identify the numbers in the peripheral circles, they click on a circle in order to verify with the help of the auditory feedback whether it was the right circle in the sequence. Thereby, they produce a high number of sequence errors. In order to perform well, participants are then forced to encode the clicking sequence explicitly. The free-gaze group, however, can use the numbers as external memory (65) to click the locations in the correct order, thereby preventing sequence errors. During the recall phase, however, also the free-gaze participants need to rely on explicit knowledge about the target sequence, which they did not had to learn in such a detail as the fix-gaze group during the visual phase. This explicit knowledge benefit helps the fix-learning participants to perform better when the recall phase starts as indicated by their now smaller amount of sequence errors. Experiment 4 used an experimental design that eliminates this presumable reason for the “fixation-learning” benefit.

## Experiment 4

In Experiments 1-3, a strong “fixation-learning” benefit could be observed immediately after having to resume the learned clicking sequence on an empty screen. This benefit might be due to forced early sequence encoding. In order to keep the need for early sequence learning at the same level across all participants, no numbers were provided within the target circles. Instead, participants had to find the right clicking sequence by trial and error with the help of auditory feedback. In this way, all participants are encouraged to encode the clicking sequence explicitly early during the visual phase, independent of whether they have to keep central fixation or are allowed to move their eyes freely. Additionally, the sample size was doubled in order to have more power to uncover a possible benefit of looking-at-nothing after the removal of the “fixation-learning” benefit.

### Methods

Eighty right-handed students (35 male and 45 female) with a mean age of 25 years completed Experiment 4.

Apparatus, stimuli, and procedure differed from Experiment 2 in two aspects. Firstly, a new configuration of target positions was generated (Figure 1, top right) with the same prerequisites as in Experiment 1. Secondly, no numbers were presented in the target circles during the visual phase, so that the participant had to learn the clicking sequence from trial and error with the help of the auditory feedback. In this way, no numbers can be used as external memory, so that also the free-gaze participants need to encode the required clicking sequence explicitly early during the acquisition phase in order to perform well. This manipulation was introduced in order to prevent any benefit from learning with central fixation for later memory-based clicking, so that a possible benefit of looking-at-nothing can be uncovered.

Analyses were the same as in the previous experiments.

### Results

#### Learning Phase

Participants of the fix-gaze group made on average 0.6 fixations outside of the fixation region per trial. Participants of the free-gaze group performed on average 6.4 guiding fixations per trial. The mixed ANOVAs (learning group x block) revealed significant block main effects for all performance measures, significant group main effects for trial completion time, number of all and precision errors, click precision, and cursor-path length, but not sequence errors and a significant interaction for completion time, sequence errors, and precision errors (Table 7).

**Figure 8.**
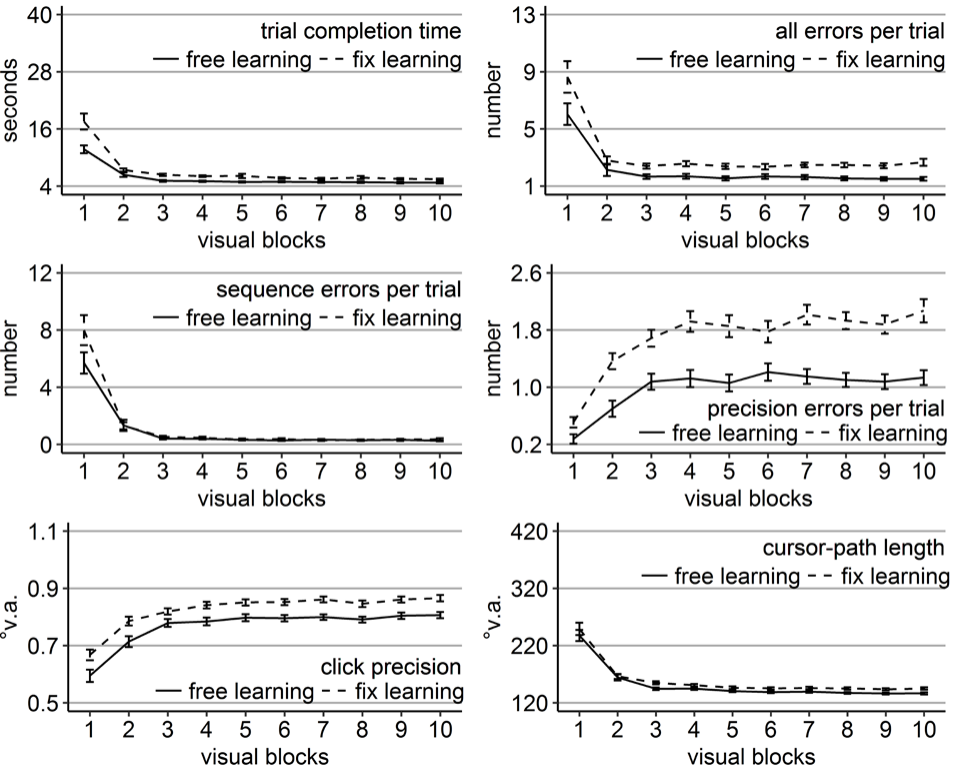
Performance measures during the learning phase of Experiment 4: Trial completion time in seconds, numbers of all errors, sequence errors, and precision errors per trial, click precision in °v.a., and cursor-path length in °v.a. (y-axes) over the course of the ten visual blocks (x-axes) for the two learning groups (free gaze as solid line and fix gaze as dashed line). Error bars represent standard errors of the mean.

As in the previous experiments, performance in terms of trial completion time, cursor-path length, and errors of all types improved significantly over the course of learning, while click precision decreased (all linear trend *p*s < .001, Figure 8). Participants who were allowed to move their eyes freely mostly completed a trial significantly faster, with shorter cursor-paths, higher click precision, and fewer precision errors than participants who had to keep central fixation (Figure 8) as confirmed by significant independent sample *t*-tests per block. Specifically, completion time differed significantly between groups in all blocks except block 2 (*t*(71.58) = 1.75, *Cohen’s d_z_* = 0.39, *p* = .08, but Wilcoxon signed rank test *W* = 448.5, *p* < .001), number of all errors in blocks 3-10 (and block 2 according to Wilcoxon signed rank test with *W* = 454.00, *p* < .001), precision errors in all blocks, cursor-path length in blocks 3 and 5-10, and click precision in all blocks. Importantly, this time groups did not differ significantly in terms of sequence errors in any of the visual blocks and the free-gaze group produced a reasonable amount of sequence errors early during learning, indicating that all participants learned from trial and error.

**Table 7:** ANOVA results of the learning phase of Experiment 4.

***DV***	***effect***	***df***	***F***	***η^2^***	***p***	***ε***
**completion time**	**L**	**1, 78**	**14.16**	**.06**	**< .001**	
	**B**	**9, 702**	**101.21**	**.47**	**< .001**	**.14**
	**L x B**	**9, 702**	**7.10**	**.06**	**< .001**	**.14**
**number of all errors**	**L**	**1, 78**	**12.74**	**.05**	**< .001**	
	**B**	**9, 702**	**59.39**	**.34**	**< .001**	**.14**
	L x B	9, 702	1.76	.01	.07	
**number of sequence errors**	L	1, 78	2.00	.00	.16	
	**B**	**9, 702**	**95.57**	**.50**	**< .001**	**.13**
	**L x B**	**9, 702**	**2.92**	**.03**	**< .01**	
**number of precision errors**	**L**	**1, 78**	**23.82**	**.18**	**< .001**	
	**B**	**9, 702**	**63.91**	**.18**	**< .001**	**.82**
	**L x B**	**9, 702**	**4.81**	**.02**	**< .001**	**.82**
**click precision**	**L**	**1, 78**	**17.93**	**.11**	**< .001**	
	**B**	**9, 702**	**92.83**	**.35**	**< .001**	**.62**
	L x B	**9, 702**	0.50	.00	.87	
**cursor-path length**	**L**	**1, 78**	**4.36**	**.02**	**< .05**	
	**B**	**9, 702**	**176.42**	**.89**	**< .001**	**.16**
	L x B	9, 702	0.31	.00	.97	

DV = dependent variable, L = learning group, B = block, df = degrees of freedom, F = test value, η^2^ = generalized eta-squared, p = significance value, ε = Greenhouse-Geisser’s epsilon. Significant effects are printed in bold.

#### Recall Phase

**Figure 9.**
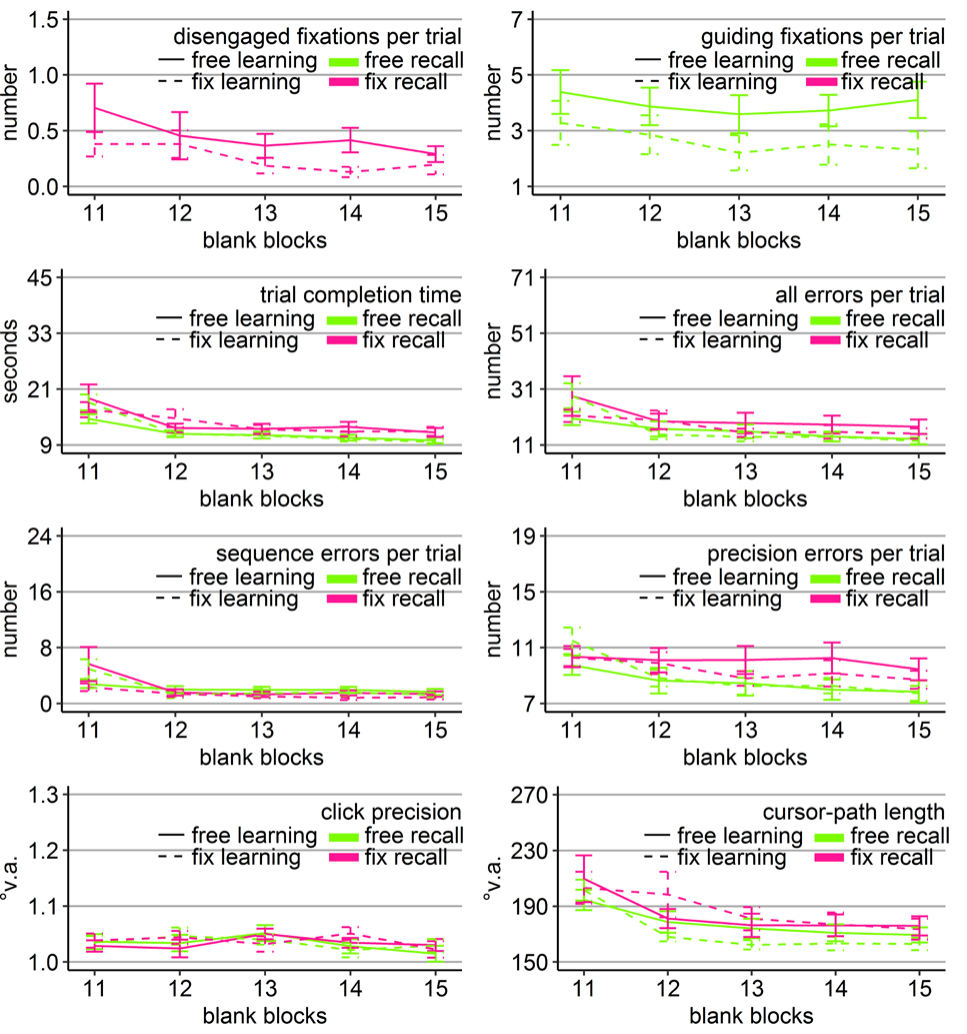
Gaze and performance measures during the recall phase of Experiment 4. The top left plot shows how often participants who were asked to keep central fixation, disengaged their fixation per trial (y-axis) over the course of the five blank blocks (x-axis) depending on whether they learned the clicking sequence with free gaze (solid line) or with fixed gaze (dashed line). The top right plot shows how many guiding fixations participants who were allowed to move their eyes freely, performed per trial (y-axis) during the five blank blocks (x-axis) depending on whether they had learned the clicking sequence with free gaze (solid line) or with fixed gaze (dashed line). The six bottom plots show the six performance measures trial completion time in seconds, numbers of all errors, sequence errors, and precision errors per trial, click precision in °v.a., and cursor-path length in °v.a. (y-axes) over the course of the five blank blocks (x-axes) for the four group combinations of recall with free gaze (green lines) or fix gaze (pink lines) after learning with free gaze (solid lines) or fix gaze (dashed lines). Error bars represent standard errors of the mean.

On average 0.4 fixations were observed outside of the fixation region per fix-gaze participant and blank-screen recall trial. The mixed ANOVA (learning group x block) did only reveal a significant block effect (Table 8) due to a significant improvement in keeping central fixation over the course of the blank-screen phase (linear trend *p* < .05; Figure 9). However, Friedman test indicated a group effect (*Χ^2^*(3) = 5, *p* < .05), but Wilcoxon signed rank test did not reveal any group differences per block.

Participants who were allowed to move their eyes freely executed on average 3.3 guiding fixations per recall trial. The mixed-design ANOVA (learning group x block) revealed only a significant block effect (Table 8), due to a slightly decreasing number of guiding fixations over the course of the blank-screen phase (linear trend *p* < .05; Figure 9). Friedman test indicated a group effect (*Χ^2^*(3) = 5, *p* < .05), presumably due to the fact that only the free-learning group contributed significantly to the overall linear trend (linear trend *p* < .05 vs. *p* = .38).

**Table 8:** ANOVA results of the recall phase of Experiment 4.

***DV***	***effect***	***df***	***F***	***η^2^***	***p***	***ε***
**disengagements**	L	1, 38	2.17	.03	.15	
	**B**	**4, 152**	**3.33**	**.04**	**< .05**	**.60**
	L x B	4, 152	0.64	.01	.64	
**guiding fixations**	L	1, 38	2.05	.04	.16	
	**B**	**4, 152**	**3.18**	**.01**	**< .05**	**.74**
	L x B	4, 152	0.60	.00	.66	
**completion time**	L	1, 76	0.03	.00	.85	
	**R**	**1, 76**	**4.03**	**.03**	**< .05**	
	**B**	**4, 304**	**29.14**	**.16**	**< .001**	
	L x R	1, 76	0.21	.00	.65	.45
	L x B	4, 304	0.50	.00	.73	
	R x B	4, 304	0.20	.00	.94	
	**L x R x B**	**4, 304**	**2.68**	**.02**	**< .05**	
**number of all errors**	L	1, 76	0.28	.00	.60	
	R	1, 76	1.34	.01	.25	
	**B**	**4, 304**	**15.66**	**.08**	**< .001**	
	L x R	1, 76	0.66	.01	.42	.41
	L x B	4, 304	0.34	.00	.85	
	R x B	4, 304	0.44	.00	.78	
	L x R x B	4, 304	2.63	.01	.03	
**number of sequence errors**	L	1, 76	0.99	.01	.32	
	R	1, 76	0.40	.00	.53	
	**B**	**4, 304**	**11.45**	**.08**	**< .001**	
	L x R	1, 76	1.18	.01	.28	.30
	L x B	4, 304	0.01	.00	1.00	
	R x B	4, 304	0.22	.00	.93	
	**L x R x B**	**4, 304**	**3.64**	**.03**	**< .05**	
**number of precision errors**	L	1, 76	0.05	.00	.82	
	R	1, 76	2.25	.02	.14	
	**B**	**4, 304**	**10.82**	**.04**	**< .001**	
	L x R	1, 76	0.66	.01	.42	.78
	L x B	4, 304	1.72	.01	.15	
	**R x B**	**304**	**2.46**	**.01**	**< .05**	
	L x R x B	4, 304	0.37	.00	.83	
**click precision**	L	1, 76	0.10	.00	.75	
	R	1, 76	0.05	.00	.83	
	B	4, 304	1.79	.01	.13	
	L x R	1, 76	0.02	.00	.88	
	L x B	4, 304	1.13	.01	.34	
	R x B	4, 304	0.89	.01	.47	
	L x R x B	4, 304	0.71	.01	.58	
**cursor-path length**	L	1, 76	0.05	.00	.82	
	R	1, 76	2.35	.02	.13	
	**B**	**4, 304**	**23.25**	**.09**	**< .001**	
	L x R	1, 76	0.38	.00	.54	.53
	L x B	4, 304	0.36	.00	.84	
	R x B	4, 304	0.37	.00	.83	
	L x R x B	4, 304	1.96	.01	.10	

DV = dependent variable, L = learning group, R = recall group, B = block, df = degrees of freedom, F = test value, η^2^ = generalized eta-squared, p = significance value, ε = Greenhouse-Geisser’s epsilon. Significant effects are printed in bold.

The mixed-design ANOVAs (block x learning group x recall group) for the performance measures revealed significant block effects for all performance measures except for click precision. In addition, there was a significant recall-group effect and a three-way interaction for trial completion time, a significant three-way interaction for sequence errors, and a significant interaction of recall group x block for precision errors (Table 8, Figure 9). Importantly, none of the learning group main effects reached significance, nor any learning group x block, nor any learning group x recall group interaction (Table 8).

The block effects were due to improving performance over the course of the recall phase, indicated by significant linear trends (time: *p* = .05 for free learning with fix recall and *p*s < .01 for all other group combinations, *p* < .01 fix recall group; all errors: *p* < .001; sequence errors: *p* = .10 for free learning with fix recall and *p*s < .05 for all other group combinations; precision errors: *p* < .001 for free recall and *p* = .05 for fix recall; path: *p* < .001).

The recall group effect indicated faster clicking on the empty target locations by those participants who were allowed to move their eyes freely compared to participants who had to keep central fixation (Figure 9). ANOVAs per block revealed that the recall groups differed significantly in terms of completion time during block 14 (*F*(1, 76) = 5.22, *η^2^* = .06, *p* < .05) and block 15 (*F*(1, 76) = 6.55, *η^2^* = .08, *p* < .05), independent of how they learned (non-significant learning group main effect and learning group x recall, all *F*s < 1). No other effect reached significance in the block ANOVAs.

### Discussion

As in the previous experiments, clicking a visual target sequence with free gaze was faster and more accurate than clicking the sequence with central fixation. By removing the target numbers during the visual phase, the “fixation learning” benefit during the blank-screen phase was successfully eliminated. This indicates that central fixation is not beneficial per se, but pushes an early explicit learning of the target sequence. This interpretation is confirmed by the sequence errors which indicate trial-and-error learning. In the previous two experiments, the fix-learning group executed sequence errors in the beginning of the visual phase because they could only locate the circles, but could not identify the numbers in the periphery. The free-learning group, however, used the numbers as external memory, so that they did not erroneously click a wrong circle in the sequence. However, when it came to the recall phase, the fix-learning group benefitted from having already learned the sequence explicitly, so that they executed less sequence errors in the blank phase compared to participants that had learned the clicking sequence with free gaze. The empty circle display in Experiment 4 forced all participants to learn from trial and error as indicated by a comparably high number of sequence errors in the beginning of the visual phase and a comparably low number of sequence errors in the recall phase. Having successfully prevented the “fixation-learning” benefit, a small looking-at-nothing benefit for trial completion time was observed, independent of how participants had learned the task, however only in two blocks. Thus, it might be that looking-at-nothing has a small functional role when having to reproduce a sensorimotor sequence in the absence of previously learned visual information.

## Experiment 5

In Experiments 1-4, it was investigated whether a looking-at-nothing benefit can be observed, when participants have to complete a sensorimotor location sequence that had been learned with the help of visual targets (numbered or empty circles). Only a very small benefit of fixating empty action target locations could be found late during the blank-screen recall phase. Therefore, looking-at-nothing might have a small facilitating effect on memory recall after a familiarization period with the blank-screen task, provided that the “fixation-learning” benefit is prevented. However, it is possible that looking-at-nothing is much more helpful for encoding non-visually marked spatial locations rather than recall of visual representations. In order to test this possibility, no visual phase was provided in Experiment 5. Instead, participants saw a blank screen from the first trial on and had to find the target locations and the correct sequence by trial and error with the help of auditory feedback. If looking-at-nothing supports encoding of non-visually marked locations, then a much stronger benefit should arise in this situation, when no visual memory representation for the target locations is present beforehand.

### Methods

Forty right-handed students (19 male and 21 female) with a mean age of 25 years completed Experiment 5. The data of one additional participant were incomplete and did therefore not enter the analyses.

Apparatus and stimuli were the same as in Experiment 3, except that no visual target information was provided throughout the experiment. A new spatial configuration of eight target regions was generated with the same prerequisites as in Experiment 1. The configuration was again the same throughout the entire experiment.

However, the experimental phase consisted of 10 blocks à 10 trials blank-screen phase, only. As no visual phase preceded the blank-screen phase, participants could solve the task only by trial and error using the auditory feedback after a correct click. A “fixation-learning” benefit is thereby again prevented. If looking-at-nothing supports memory recall of previously visible information, no free-gaze benefit should arise this time. If looking-at-nothing supports memory encoding of non-visually marked locations, a much stronger free-gaze benefit should arise here, most notably during later blank blocks. If looking-at-nothing supports memory recall of locations independent of how they were learned (visual vs. non-visual), then a late, comparably weak looking-at-nothing benefit should arise as in the previous experiment.

Analyses were the same as in the previous experiments.

### Results

#### Blank-screen Phase

**Figure 10.**
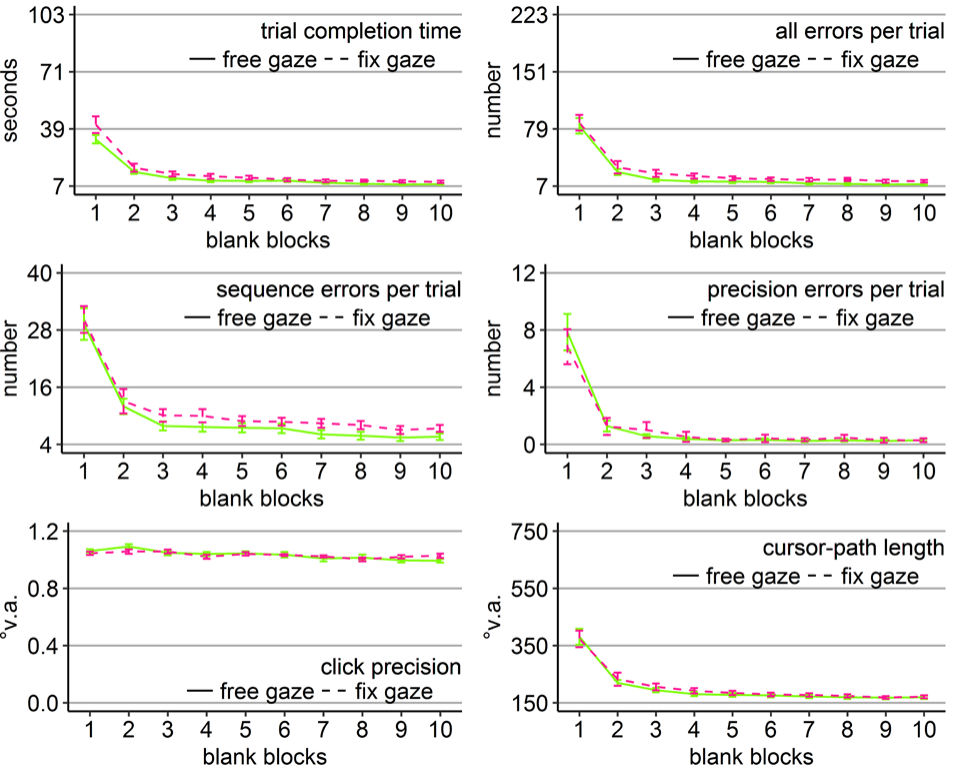
Performance measures during Experiment 5: Trial completion time in seconds, numbers of all errors, sequence errors, and precision errors per trial, click precision in °v.a., and cursor-path length in °v.a. (y-axes) over the course of the ten blank-screen blocks (x-axes) for the two groups (free gaze as pink solid line and fix gaze as green dashed line). Error bars represent standard errors of the mean.

Participants of the fix-gaze group made per trial on average 0.5 fixations outside of the fixation region. Participants of the free-gaze group performed on average 4.6 guiding fixations per trial. Mixed-design ANOVAs with blank block (1-10) as within-subject factor and gaze group (free vs. fix) as between-subject factor for all performance measures revealed significant block effects, no group effects, and no interactions (Table 9 and Figure 10). However, there were interaction trends for trial completion time and click precision and significant Friedman tests for the number of all errors (*Χ^2^*(3) = 10, *p* < .01) and sequence errors (*Χ^2^*(3) = 10, *p* < .01).

Block effects were due to improving performance over the course of the blank-screen phase, indicated by significant linear trends (*p*s < .001). Independent sample *t*-tests per block for trial completion time revealed that the block x group interaction trend was due to significantly faster clicking by the free-gaze group in block 8 (*t*(36.84) = 2.17, *Cohen’s d_z_* = .69, *p* < .5) and block 9 (*t*(25.68) = 2.18, *Cohen’s d_z_* = .69, *p* < .5). There were no significant group differences for all other measures and blocks, neither in parametric nor non-parametric testing.

**Table 9:** ANOVA results of the recall phase of Experiment 5.

***DV***	***effect***	***df***	***F***	***η^2^***	***p***	***ε***
**completion time**	G	1, 38	2.60	.03	.11	
	**B**	**9, 342**	**118.15**	**.61**	**< .001**	**.17**
	G x B	9, 342	1.77	.02	.07	
**number of all errors**	G	1, 38	1.57	.02	.21	
	**B**	**9, 342**	**96.80**	**.58**	**< .001**	**.17**
	G x B	9, 342	0.12	.00	1.00	
**number of sequence errors**	G	1, 38	1.37	.02	.25	
	**B**	**9, 342**	**85.99**	**.52**	**< .001**	**.22**
	G x B	9, 342	0.12	.00	1.00	
**number of precision errors**	G	1, 38	0.00	.00	.99	
	**B**	**9, 342**	**52.48**	**.51**	**< .001**	**.16**
	G x B	9, 342	0.39	.01	.94	
**click precision**	G	1, 38	0.00	.00	.98	
	**B**	**9, 342**	**7.26**	**.10**	**< .001**	**.67**
	G x B	9, 342	1.64	.02	.10	
**cursor-path length**	G	1, 38	0.18	.00	.67	
	**B**	**9, 342**	**96.35**	**.56**	**< .001**	**.17**
	G x B	9, 342	0.22	.00	.99	

DV = dependent variable, G = gaze group, B = block, df = degrees of freedom, F = test value, η^2^ = generalized eta-squared, p = significance value, ε = Greenhouse-Geisser’s epsilon. Significant effects are printed in bold.

A between-subject ANOVA with experiment (4 vs. 5) and gaze group (free vs. fix gaze or respectively free vs. fix recall) on the mean trial completion time of blocks 14 and 15 of Experiment 4 and blocks 8 and 9 of Experiment 5 did not reveal a significant interaction, arguing that the size of the recall effect was not significantly different across the two experiments (group: *F*(1, 116) = 9.70, *η^2^* = .08, *p* < .01; experiment: *F*(1, 116) = 5.77, *η^2^* = .05, *p* < .05; *F*(1, 116) = 0.04, *η^2^* = .00, *p* = .84).

### Discussion

In Experiment 5, participants had 100 trials to learn in a trial-and-error fashion from auditory feedback where invisible targets were located on the computer screen and in which sequence they had to be clicked. Although numerically slightly worse, performance measures of the group that learned the invisible target sequence with central fixation did mostly not differ significantly from the group that learned with unrestricted gaze. A very small completion-time benefit arose during later blank-screen blocks, however, the difference was not stronger than in the previous experiment. The results argue against a functional role of looking-at-nothing for motor control or for encoding non-visually marked locations. Otherwise benefits would have been stronger here than in the previous experiment and also constant over the course of the blank-screen phase.

## General Discussion

In the present study, I investigated the function of looking-at-nothing for sensorimotor control. In four experiments, participants completed 100 sequential clicking trials with constant visible targets on a computer screen. Afterwards, they were asked to click the same location-sequence on a blank screen for several blocks. In a fifth experiment, participants had to learn the clicking sequence on a blank screen throughout the experiment. Half of the participants were not restricted in their eye movements, while the other half had to keep central fixation throughout each trial during the visual phase as well as counter-balanced during the blank-screen phase. Replicating and extending previous findings (18,19,20), performance measured as trial completion time, errors, click precision, and cursor-path length was significantly better when participants were allowed to look at the visual targets, also in a task without direct target-effector mapping, i.e., a task in which the hand moves a mouse on the table controlling the movements of a cursor on a computer screen. This result is in line with studies indicating that computer mouse actions are comparable to real-world manual actions (22,66).

When visual information was no longer available, participants who were allowed to move their eyes spontaneously fixated on the target locations sequentially, a behavior called looking-at-nothing (22,26,67). Unexpectedly, task performance in the recall phase benefitted strongly from having learned with central fixation, provided that the sequence had been indicated by numbers (Experiments 1-3). If the sequence had to be learned from trial and error (empty circles), this “fixation-learning” benefit was eliminated (Experiment 4). While free-gaze participants could use the numbers in Experiments 1-3 as external memory (65), fix-gaze participants could not use this strategy because they could not identify the numbers in the periphery as indicated by a high amount of initial sequence errors. By erasing the numbers, both groups were forced to learn the clicking sequence explicitly enhancing the performance of the free-learning group to the fix-learning level during the recall phase.

A benefit of looking-at-nothing could only be found, when it was no longer overshadowed by the “fixation-learning” benefit. Specifically, a free-gaze benefit was found late during the blank-screen phase, when the “fixation-learning” benefit had already washed out (Experiment 3) or when the “fixation-learning” benefit was experimentally eliminated (Experiments 4 and 5). However, this benefit of looking-at-nothing was very small and fragile. The following discussion will elaborate why humans might look at target locations normally and in the absence of visual information.

### Why do we look at target locations?

When visual information is available, important features of an action target such as its orientation, surface, and size can be extracted by a fixation that brings these important features on the fovea, the area on the retina with the highest spatial resolution. Extracting these features by so-called guiding fixations (cf. 3,4,18,19,20,50) is important to calculate the needed hand-movement parameters such as direction, force, and grip aperture (11, 13). Thus, it is not surprising that there was a performance benefit in terms of trial completion time, number of errors, click precision, and cursor-path length of those participants who were allowed to move their eyes freely compared to those participants who had to keep central fixation when acting on visual targets, as the latter had to rely on target information extracted from the visual periphery with lower spatial resolution.

However, here and in previous studies (22,26,67,68) it was observed that participants spontaneously fixate empty target locations and have trouble to omit this behavior if asked to keep central fixation (observed disengagements). It had been speculated that the spontaneous looking-at-nothing behavior can be observed because it has a functional role in task execution (29,38,39,69), e.g., by facilitating motor calculation, memory encoding, or memory recall. However, in the present study, the benefit of looking-at-nothing was very small and only apparent under certain circumstances. Specifically, looking-at-nothing improved performance compared to central fixation only when participants were already familiarized with the sensorimotor sequence.

### Why was the benefit of looking at empty target locations so small?

In a precursor study (22), it was investigated how robustly participants scan remembered target locations in a sensorimotor task. Participants had to click the same sequence of nine numbered circles as fast as possible for 50 trials in a learning phase. Thereafter, they had to complete the same sequence on an empty computer screen, again for 50 trials. It was found that participants sequentially scanned the visual as well as the empty target locations indicated by a similar amount of guiding fixations and highly similar scan paths. However, their fixations were more distant from the center of each target location in case of empty instead of visually marked locations. This indicates that target locations stored in long-term memory come with a certain imprecision. Thus, using the current gaze position as a deictic pointer for the effector movement (2,38) might not be useful and could be the reason why looking-at-nothing did not seem to have any functional role for motor calculation in the present study.

The precursor study (22) also revealed that the looking-at-nothing behavior is intensified when repeatedly having to act on empty target locations. Thus, when unexpectedly asked to act on empty target locations from memory, looking-at-nothing is not at its maximum, so that its possible benefit will also be undermined. This is in line with the present finding that a free-gaze benefit arises, if at all, late during the empty-screen phase.

In addition, the precursor study (22) found that participants did not only look at empty target locations shortly before they act on them (guiding fixations), they also re-fixated on target locations that had already been successfully acted on (checking fixations, cf. 7,22,55). Presumably, these checking fixations were used to refine the long-term memory representation of the target locations. This indicates that explicit memory for the target locations also needed improvement when the blank-screen phase started. As the learning phase consisted of numbered circles, participants were not forced to encode the target location sequence explicitly, but could use the numbers as external memory (65). The same was true in Experiments 1-3, so that when it came to acting on empty target locations, the representation of the location sequence might have been too worse in order to benefit from looking-at-nothing. However, even in Experiment 4, when explicit sequence encoding was forced by having to learn the correct sequence of empty circles by trial and error, looking-at-nothing did not constitute a large benefit.

It has to be noted that covert attention can be shifted sequentially to the target locations in case of central fixation and these shifts of covert attention might already be enough for successful motor guidance (cf. 34) for the same argumentation for the function of looking-at-nothing in a memory-recall task).

### Is looking-at-nothing functional at all?

Looking-at-nothing was originally reported in visual imagery and memory recall tasks (23,24,25,26 27 28,29,30,31,32). From these tasks, there is evidence that eye movements to empty locations can facilitate remembering previously presented material. When recalling disappeared visual information, fixating on the corresponding location can help to remember the information and to rebuild a mental image (26,27,28,29,31,36,37,70,71,72). In addition, it seems that shifting the eyes during recall in a similar way as during encoding also helps to extract more information with higher quality (69). When forced to fixate on a different location than that location where a to-be-remembered item was originally positioned, recall is impaired (26). Especially the spatial relationship between memory items seems to be recalled easier when fixating on the remembered locations (72). Thus, looking-at-nothing seems to support memory recall in explicit memory tasks.

Could looking-at-nothing support sensorimotor control by the same memory-recall mechanism? Shifting the eyes to the current target location could help to recall the features of the current target such as its size and orientation and also the location of the following target in the sequence. In this way, effector movements could be adapted for the current sub-action, while attention could already be shifted towards the next target location. The results of the present study are partly in line with a memory-recall function of looking-at-nothing for sensorimotor tasks. Namely, the fact that benefits of looking-at-nothing arose late during the blank-screen phase when explicit memory should be best, could be interpreted as support for a memory-recall function. Thus, there might be a unitary memory-recall mechanism of looking-at-nothing for different tasks.

Although there was a looking-at-nothing benefit in some experiments, this effect should not be overestimated, not only because it was very small and fragile, but also because keeping central fixation, the control condition, might be costly in itself. Thus, alternatively, looking at remembered action-target locations might rather be a by-product of learning and automatization in highly-practiced sensorimotor sequences, while it has a real function only in tasks with more emphasis on explicit memory recall (see cited evidence above). As looking-at-nothing supports recall of memorized visual details, future studies have to investigate whether looking-at-nothing might have more pronounced effects on sensorimotor tasks that depend on the quality of the memory representation of the action targets, e.g., with small-sized targets, high-precision instruction, or object-dependent action selection.

It is also possible that sensorimotor tasks with direct eye-hand mappings, i.e., when target position of gaze and hand match, benefit more from looking-at-nothing. The benefit from deictic codes (2,38) might even be restricted to this case, in which the fixation point in the world directly determines the hand-target position, rather than the target position of a mouse cursor that is moved by the hand on a table in a different plane (horizontal movements on the table translated into vertical movements of the cursor on the computer screen).

In addition, it has been argued that there are similar memory benefits of shifting the eyes and shifting covert attention to remembered locations (34, 73,74,75). Thus, the mechanisms by which looking-at-nothing can be beneficial for memory recall may likely be attention allocation. During central fixation covert shifts of attention to empty target locations are possible. Thus, future studies have to investigate whether there is a stronger motor performance difference with instructed eye movement sequences that are either compatible or incompatible with the target location sequence (cf. 26). This procedure could also investigate whether the small looking-at-nothing benefit found here was in reality exclusively due to the cost of keeping central fixation.

### What constitutes the benefit of learning with central fixation?

Interestingly and unexpectedly, there was a strong and robust performance benefit during acting on remembered target locations of participants who had learned the visuospatial sequence with central fixation instead of unrestricted gaze. Why is a learned clicking sequence reproduced more accurately and faster on a blank screen, when it was learned with central fixation rather than with unrestricted eye movements and thus target fixations?

There are in principle four possible reasons for a “fixation-learning” benefit. Firstly, it is possible that a retinotopic map of the target configuration is acquired when having to keep central fixation throughout the clicking trials. A retinotopic map is a map where locations are coded on the basis of their position on the retina (76). Such an additional representation of the target locations could help to remember the target locations better on the blank screen compared to a spatiotopic representation alone (77).

Secondly, an explanation with similar predictions is that participants benefit from central fixation, because they do not have to shift the frame of reference in-between clicks. The reference for their movements is always the central fixation cross and their gaze position on it. However, these explanations as well as the retinotopic map explanation would predict a stronger “fixation-learning” benefit for participants who continue central fixation than for participants who move their eyes freely during recall. In addition, the reference frame explanation predicts an increase in precision errors over the course of learning only for the free-gaze group due to the accumulated error from realigning the frame of reference. Contrastingly, precision errors increased for both groups, and central fixation rather than free gaze caused more precision errors during learning (Experiments 2-4).

Thirdly, participants who learn with central fixation are significantly slower in clicking the visual target sequence, which prolongs their exposure to the to-be-learned sequence. Even if they can only view the numbered circles peripherally, the extended exposure time might give them an encoding benefit. Additionally, the fix-learning participants spare time for saccade planning and execution, which might be traded for encoding.

Fourthly, when having to keep central fixation, the targets have to be located and identified from peripheral vision. Object identification in the periphery is restricted due to its low resolution (62, 63,64). In the present study, identification of the numbers in the periphery when having to keep central fixation is hard, as indicated by the high amount of sequence errors. Therefore, the central-fixation group is forced to explicitly encode the clicking sequence early during the learning phase in order to reduce sequence errors and gain speed. The free-gaze group, however, can use the numbers as external memory (65) to click the locations directly in the right order as indicated by the absence of sequence errors. When it comes to acting on the blank screen, however, the free-learning group lacks the external location memory and is thus outperformed by the fix-learning group independent of whether the recall phase demands central fixation or allows free gaze (not only FixFix is better, but also FixFree is better than FreeFix and better than FreeFree).

The design of Experiment 4 forced also the free-gaze group to encode the number sequence explicitly, while leaving intact all other possible fixation advantages. Specifically, by using empty target circles, all participants had to find the correct clicking sequence from trial and error on the basis of the auditory feedback. In this way, participants were forced to explicitly encode the clicking sequence to perform the task regardless of whether they had to keep central fixation or were allowed to move their eyes freely. Indeed, this manipulation increased the number of sequence errors during learning made by the free-learning group to the level of the fix-learning group. More importantly, the manipulation successfully eliminated the “fixation-learning” benefit during recall, indicating that the benefit was primarily due to enforced explicit encoding. The data do not support the idea that learning via a retinotopic map or the usage of a constant reference frame is beneficial.

### Summary

In the present study, it was investigated whether spontaneous looking-at-nothing behavior is beneficial for performing a sequential sensorimotor task. In order to test this, participants could either move their eyes unrestrictedly or had to keep central fixation while clicking a previously learned or unknown location-sequence on a blank computer screen. Results revealed only a very small and fragile benefit of the spontaneously performed fixations on the empty target locations. As this benefit of “looking-at-nothing” appeared only late during the blank-screen phase, it constitutes most likely, if any, a memory benefit rather than a benefit for motor calculation. Although there was a looking-at-nothing benefit in some experiments, this effect should not be overestimated, not only because it was very small and fragile, but also because keeping central fixation, the control condition, might be costly in itself. Interestingly, however, central fixation can lead to a strong benefit for sequence learning by forcing participants to explicitly encode the target sequence rather than relying on external visual information.

## Ethics and Conflict of Interest

The author declares that the contents of the article are in agreement with the ethics described in http://biblio.unibe.ch/portale/elibrary/BOP/jemr/ethics.html and that there is no conflict of interest regarding the publication of this paper.

## Acknowledgements

This research was supported by the Cluster of Excellence Cognitive Interaction Technology ‘CITEC’ (EXC 277) at Bielefeld University, which is funded by the German Research Foundation (DFG).

I acknowledge support for the Article Processing Charge by the Deutsche Forschungsgemeinschaft and the Open Access Publication Fund of Bielefeld University.

I would like to thank Werner X. Schneider for providing helpful comments on this article.

## Author note

Correspondence to

Rebecca M. Foerster

Center for Interdisciplinary Research (ZiF) & Neuro-cognitive Psychology, Psychology Department & Cognitive Interaction Technology – Center of Excellence (CITEC)

Universität Bielefeld

P.O. Box 10 01 31

33501 Bielefeld

Germany

++49 (0)521 106 – 4503 (Phone)

++49 (0)521 106 – 156934 (Fax)

rebecca.foerster@uni-bielefeld.de

ORCID: 0000-0002-4632-1382
